# Intrinsic and extrinsic actions of human neural progenitors with SUFU inhibition promote tissue repair and functional recovery from severe spinal cord injury

**DOI:** 10.1038/s41536-024-00352-4

**Published:** 2024-03-22

**Authors:** Yong-Long Chen, Xiang-Lan Feng, Kin-Wai Tam, Chao-Yang Fan, May Pui-Lai Cheung, Yong-Ting Yang, Stanley Wong, Daisy Kwok-Yan Shum, Ying-Shing Chan, Chi-Wai Cheung, Martin Cheung, Jessica Aijia Liu

**Affiliations:** 1 Department of Anaesthesiology, School of Clinical Medicine, Li Ka Shing Faculty of Medicine, The University of Hong Kong, Hong Kong, China; 2 School of Biomedical Sciences, Li Ka Shing Faculty of Medicine, The University of Hong Kong, Hong Kong, China; 3 Hong Kong sanatorium hospital, Hong Kong, China; 4 Present Address: Department of Neuroscience, Tat Chee Avenue, City University of Hong Kong, Hong Kong, China

**Keywords:** Spinal cord diseases, Neural stem cells

## Abstract

Neural progenitor cells (NPCs) derived from human pluripotent stem cells(hPSCs) provide major cell sources for repairing damaged neural circuitry and enabling axonal regeneration after spinal cord injury (SCI). However, the injury niche and inadequate intrinsic factors in the adult spinal cord restrict the therapeutic potential of transplanted NPCs. The Sonic Hedgehog protein (Shh) has crucial roles in neurodevelopment by promoting the formation of motorneurons and oligodendrocytes as well as its recently described neuroprotective features in response to the injury, indicating its essential role in neural homeostasis and tissue repair. In this study, we demonstrate that elevated SHH signaling in hNPCs by inhibiting its negative regulator, SUFU, enhanced cell survival and promoted robust neuronal differentiation with extensive axonal outgrowth, counteracting the harmful effects of the injured niche. Importantly, SUFU inhibition in NPCs exert non-cell autonomous effects on promoting survival and neurogenesis of endogenous cells and modulating the microenvironment by reducing suppressive barriers around lesion sites. The combined beneficial effects of SUFU inhibition in hNPCs resulted in the effective reconstruction of neuronal connectivity with the host and corticospinal regeneration, significantly improving neurobehavioral recovery in recipient animals. These results demonstrate that SUFU inhibition confers hNPCs with potent therapeutic potential to overcome extrinsic and intrinsic barriers in transplantation treatments for SCI.

## Introduction

Traumatic spinal cord injury (SCI) is a devastating condition that causes progressive loss of neurons and oligodendrocytes, resulting in irreversible axonal damage and defective locomotion and somatosensory function. Unfortunately, there are currently no effective clinical management and treatment regimens for SCI patients, who often experience lifelong disabilities. Following injury, the limited intrinsic regenerative ability of neurons in the adult mammalian central nervous system(CNS) and absence of neurotrophic support in the extrinsic injury environment, making it difficult to restore spinal composition and architectures^1^. Exogenous grafts with therapeutic cell populations have shown promise to compensate for the loss of the damaged nervous system and improve functional recovery after SCI. In most studies, neural progenitor cells (NPCs) embedded in a fibrin-thrombin matrix containing neurotrophic factors are required to enable the survival and differentiation of grafts at the injury sites^2–4^. However, the therapeutic effects are often limited by the prolonged maturation process of hNPCs and the hostile niche that promotes grafts differentiation into astrocytes instead of neurons^3–6^. Additionally, SCI leads to the formation of glial scar, which creates a barrier-like structure, preventing regenerated axons from penetrating and projecting across the lesion site^1^. Therefore, genetically modified hNPCs with enhanced survival and neuronal differentiation properties, as well as the capability to modulate the host environment may provide a more effective repair process for traumatic SCI.

The Sonic Hedgehog(Shh) signaling pathway plays essential roles in neurodevelopment, contributing to the renewal and survival of NPCs, neuronal differentiation, axon growth and cell fate specification of motoneurons and oligodendrocytes^7,8^. SHH activation using small molecules(e.g., purmorphamine) is required for generating therapeutic cell types from human pluripotent stem cells(hPSCs) for SCI grafts, including NPCs, motoneurons, and oligodendrocytes. Moreover, transplantation of both motoneurons and oligodendrocytes into a rat SCI model led to better locomotor recovery than transplanting individual cell types^8^. In SCI, SHH is often downregulated and suppressed by reactive astrocytes around the lesion site^9^. Recent studies have revealed neuroprotective and regenerative features of administrating SHH after CNS injuries, which enhances axonal outgrowth, counteracts apoptosis, and modulates the microenvironment via promoting neurotrophic factor secretion^10–13^, suggesting that genetic manipulation of SHH signaling components can be an effective strategy in treating SCI. The suppressor of fused(SUFU) is a negative regulator of SHH signaling but exhibits a distinct mode of action compared to other SHH inhibitors. In the absence of SHH, SUFU forms inhibitory complexes with Gli1–3 transcriptional effectors of SHH signaling in the cytoplasm and promotes the proteolytic cleavage of the full-length Gli3 activator(Gli3A190) into the Gli3 repressor (Gli3R83)^14^. Dissociating the SUFU-GLI complex following SHH stimulation allows the GLI proteins to be translocated into the nucleus and converted to transcriptional activators. Previous studies showed that Sufu^−/−^ embryos exhibited expanded Shh production in the neural tube and increased expression of Olig2, a maker for the progenitor of both motoneurons and oligodendrocytes that is normally induced by Shh signaling^15^. In vertebrate development, specific deletion of *Sufu* in neural/neural crest progenitors resulted in the early onset of neuronal or glial differentiation, depending on the cellular context^16–19^. These findings raise the possibility that inactivating SUFU in hNPCs could exert beneficial effects on the injured spinal cord via SHH activation.

In this study, we have engineered hPSCs derived NPCs with SUFU inhibition. These modified NPCs exhibited enhanced survival, neuronal differentiation with extensive neurite outgrowth, and the ability to generate oligodendrocytes, counteracting the harmful effects from the injured niche in vitro and in vivo. Importantly, these modified NPCs were able to exert non-cell autonomous effects by secreting SHH proteins, enhancing the survival and neurogenesis of non-modified NPCs in vitro. Upon grafting, hNPCs with SUFU inhibition could modulate the injured microenvironment and host cells, which dramatically reduced suppressive barriers derived from glial scar around lesion sites. Notably, we observed a significant increase in the number of mature neurons with different subtypes around *SUFU KD* grafts, indicating the role of modified grafts in preventing progressive apoptosis/loss or/and promoting differentiation of host cells. The combined beneficial effects of hNPCs with SUFU inhibition resulted in more effective integration without requiring a supportive matrix, enhancing the repair of spinal cord lesions in a post-injury hostile milieu and improving locomotor function. Our findings represent a new approach to constructing genetically modified hNPCs for the treatment of SCI in both a cell-intrinsic and -extrinsic manner.

## Results

### Generation and characterization of *SUFU knockdown(KD)* hNPCs derived from human pluripotent stem cells

**Fig. 1:**
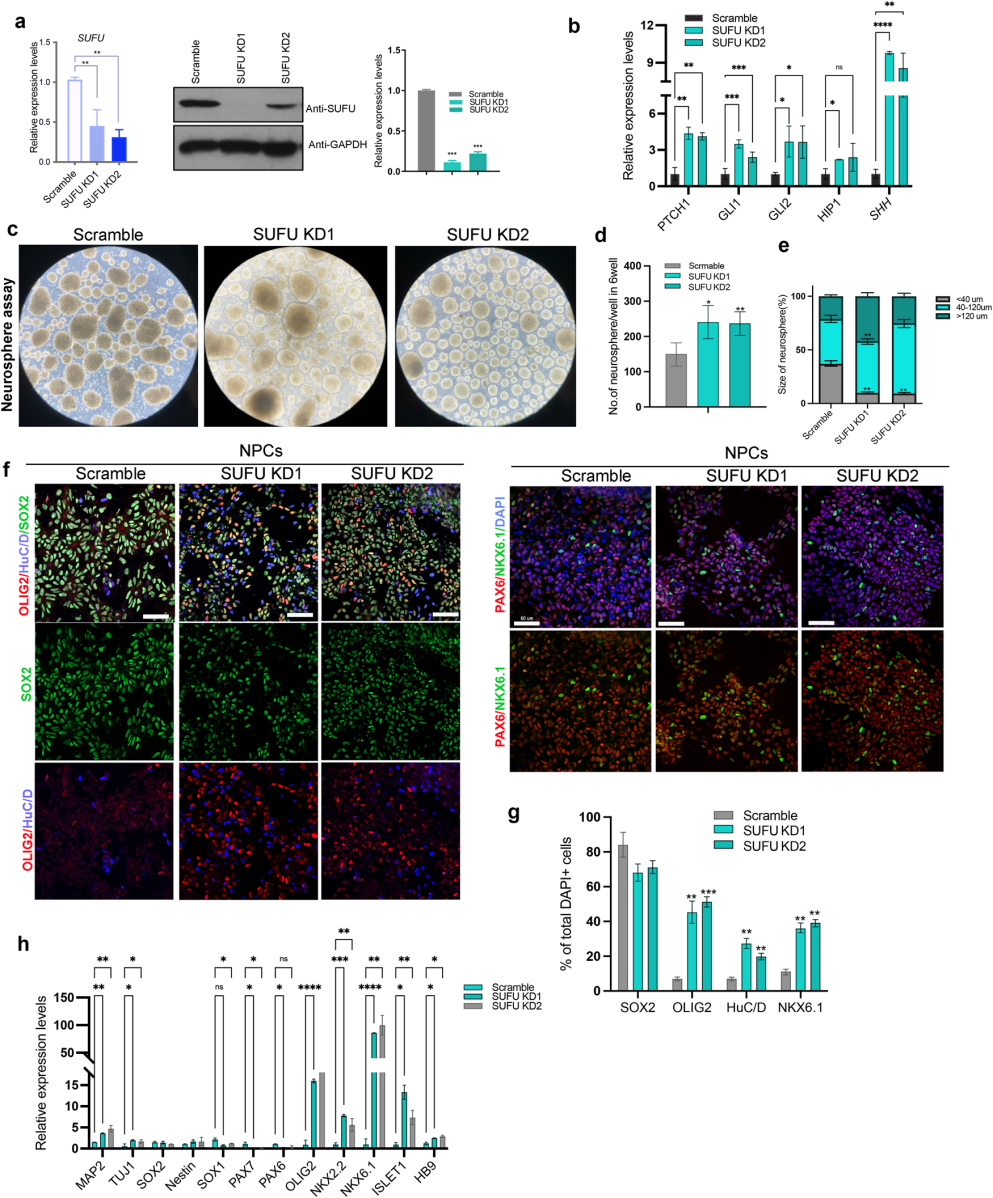
Molecular characterization of *SUFU KD* hNPCs. **a** mRNA and protein expression levels of *SUFU* in hNPCs analyzed by qPCR and immunoblotting in scramble and *SUFU KD* treatment groups. **b** qPCR analysis of *SHH* and its effectors. One-way ANOVA followed by Tukey’s post-hoc test. **c** Representative bright-field(BF) images showing the formation and renewal of neurospheres in scramble and *SUFU KD* treatment groups. Quantification of the number (**d**) and size (**e**) of neurospheres from scramble and *SUFU KD* treatment groups, One-way ANOVA followed by Tukey’s post-hoc test. Scale bar = 100 μm. **f** Representative immunofluorescence images for OLIG2, SOX2, HuC/D, PAX6, and NKX6.1 in hNPCs from scramble and *SUFU KD* treatment groups. DAPI was used as a nuclei marker. Scale bar = 50 μm. **g** Quantification of (f). **h** qPCR analysis of dorsal-ventral markers (*PAX6*,*OLIG2*,*PAX7*), neural genes (*SOX2/1*, *Nestin*) and neuronal markers (*MPA2,TUJ1,ISL1/2*, and *HB9*) in hNPCs from scramble and *SUFU KD* treatment groups, One-way ANOVA followed by Tukey’s post-hoc test. All data are expressed as mean ± SEM. Three independent experiments.

SUFU is a highly conserved negative regulator of the SHH signaling pathway. Studies have shown that deletion or inhibition of SUFU increases SHH activity, promoting the expression of Olig2, a key regulator of motoneuron and oligodendrocytes differentiation^15^, and leading to an early onset of neurogenesis or glial differentiation with transiently enhanced proliferation in the CNS or PNS^16–19^. These findings suggest that the inactivation of SUFU could trigger intrinsic specification and differentiation programs without relying on extrinsic signaling. This prompted us to investigate whether genetic manipulation of SUFU levels in hPSCs-derived NPCs could trigger the precocious acquisition of therapeutic cell types to enhance their regenerative potential for treating SCI. To address this issue, we established an in vitro differentiation protocol as described previously^20^ (Supplementary Fig. 1a) to examine the transcript levels of SUFU and SHH effectors *GLI1, PATCH1*, and *HIP1* during neural induction from three hPSCs lines. We used IMR90 for the rest of the studies, although three cell lines displayed similar results. The qPCR results showed that reduced *SUFU* expression coincided with upregulated SHH target gene expressions, indicating the elevation of SHH signaling in hNPCs during differentiation when compared with hiPSCs and embryoid body(EB)(Supplementary Fig. 1b). These findings suggest that reduced SUFU expression and activation of SHH signaling coincide with the acquisition of hNPC fate. To further explore the link between the reduction of SUFU levels and the specification/differentiation potential of hNPCs, we infected three hPSCs lines with two lentiviral-mediated short hairpin RNAs targeting *SUFU* (*SUFU KD1 and KD2)* or scramble as control by lentivirus(Fig. 1a and Supplementary Fig. 1d). Consistent with the inhibitory role of SUFU in SHH signaling, the expressions of SHH and its downstream targets (*GLI1, GLI2, PATCH1* and *HIP1)* were significantly upregulated in *SUFU KDs* hiPSCs compared to their expression levels in scramble and wild-type (WT) hiPSCs(Supplementary Fig. 1c–e). However, *SUFU KD* hiPSCs with activated SHH pathway exhibited similar expression levels of pluripotent stem cell markers compared to scramble and WT hPSCs (Supplementary Fig. 1f). In addition, EdU incorporation and annexin V apoptosis assays showed no effects on proliferation and viability in *SUFU KD* hiPSCs(Supplementary Fig. 1g). These results are consistent with previous studies^21^ showing that activation of SHH signaling does not affect pluripotency, proliferation, and survival of hPSCs.

Next, we evaluated the effects of SUFU inhibition in hNPCs. Consistently, both *SUFU KD1* and *KD2* hNPCs(passage <5) exhibited significantly reduced SUFU mRNA and protein expressions concomitant with an upregulation of SHH target genes compared to scramble control (Fig.1a, b). We further examined whether reduced SUFU expression levels could affect the self-renewal capacity of hNPCs using a neurosphere assay in the absence of growth factors, EGF and FGF. We found that secondary neurospheres(P2) from *SUFU KDs* group exhibits more spherical and transparent multicellular complexes compared to scramble control(Fig. 1c, d). Importantly, SUFU inhibition significantly increased the number of neurospheres and reduced the formation of small-sized (<40μm) neurospheres (Fig. 1d, e). We next examined the impacts of SUFU inhibition on the fate of hNPCs. Compared with the scramble group, *SUFU KD1* and *KD2* hNPCs had much higher proportions of cells expressing ventral neural progenitor marker NKX6.1, motor neuron/oligodendrocytes progenitor marker OLIG2, and pro-neuronal marker HuC/D with subtle reduction of SOX2 that could be due to accelerated neurogenesis(Fig. 1f, g). Consistently, qPCR analysis revealed significant upregulated ventral neural patterning genes *(NXK2.2, NKX6.1*, and *OLIG1/2*) and motor neuronal genes(*Tuj1, HB9*, and *ISLET1/2*), along with downregulation of neural plate border markers *PAX7*(Fig. 1h). These data indicate that *SUFU KD* hNPCs exhibited enhanced activation of SHH signaling with the acquisition of pro-neurogenic potency bias toward motor neurons and oligodendrocyte lineages.

### SUFU inhibition triggers a spontaneous differentiation program to promote the formation of motoneurons and oligodendrocytes

**Fig. 2:**
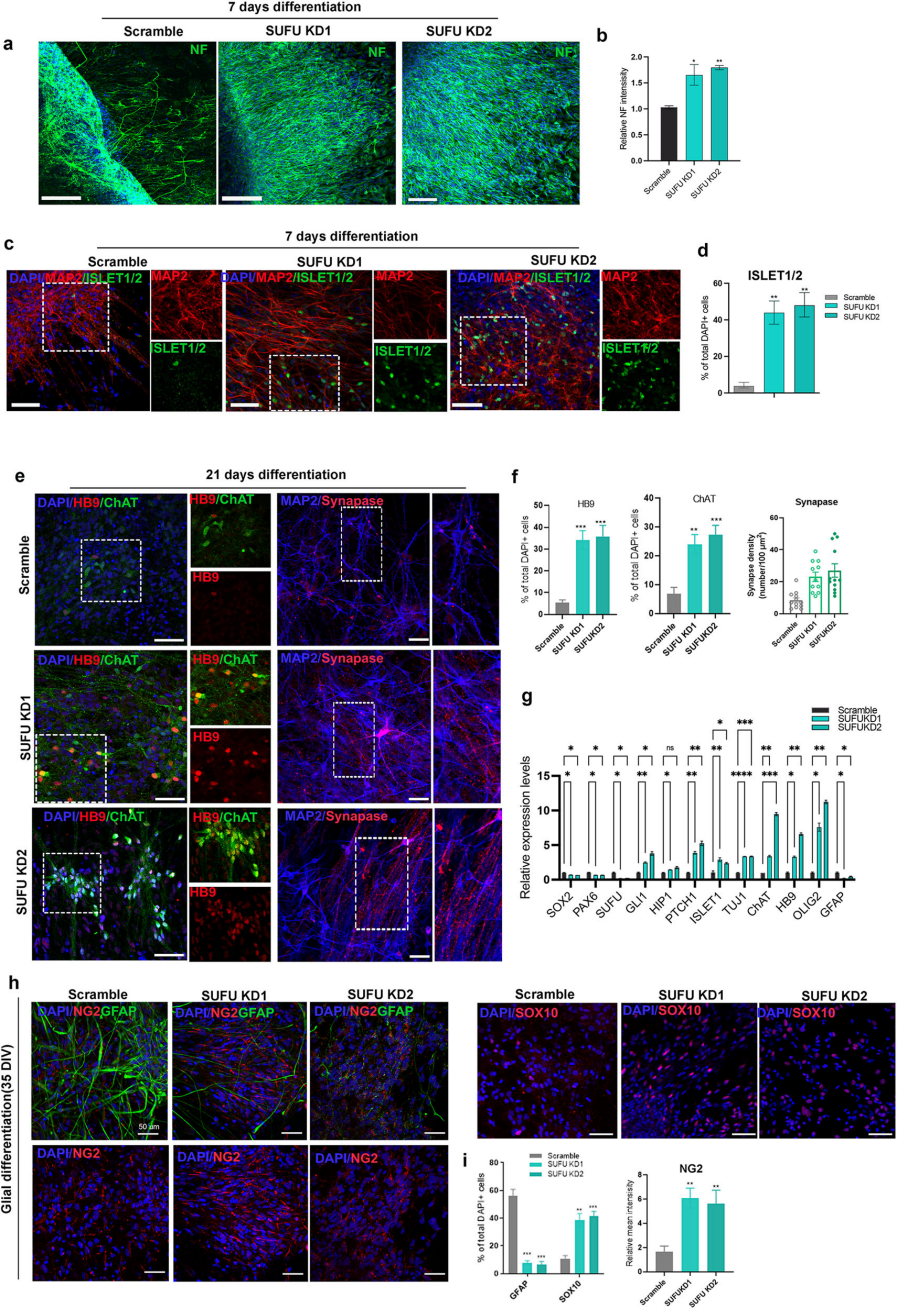
hNPCs with SUFU inhibition exert cell intrinsic differentiation capacity in generating neurons and oligodendrocytes. **a** Representative immunofluorescence of neurofilament(NF) from scramble and *SUFU KDs* neurospheres at 7 days differentiation in the absence of neurotrophic factors. Scale bar = 100 μm. **b** Quantification of relative intensity of axon outgrowth in scramble and *SUFU KDs* neurosphere. **c** Representative immunofluorescence images of MAP2, and ISL1/2 in scramble and *SUFU KD* hNPCs at 7 days differentiation. The white box shows a magnified view with indicated markers. Scale bar = 100 μm. DAPI was used as a nuclei marker. **d** Percentage of ISL1/2-positive cells in scramble and *SUFU KDs* hNPCs after 14 days differentiation. **e** Representative immunofluorescence images of ChAT, HB9, MAP2, and synaptophysin (SYN) in scramble and *SUFU KDs* hNPCs at 21 days differentiation. The white box shows a magnified view with indicated markers, Scale bar = 100 μm. DAPI was used as a nuclei marker. **f** Quantification of (**e)**. **g** qPCR analysis of HH effectors (*SUFU*, *GLI1*, and *PATCH1*), neural genes (*SOX2* and *PAX6*) and neuronal markers (*MPA2,TUJ1,ISL1/2*, *ChAT*, and *HB9*) at 21 days differentiation of scramble and *SUFU KDs* treatment groups. **h** Representative immunofluorescence images of GFAP, NG2 and SOX10 after 3 weeks differentiation in scramble and *SUFU KDs* hNPCs, Scale bar = 100 μm. **i** Quantification of (**h**). Student’s *t* test. All data are expressed as mean ± SEM. *p < 0.01, **p < 0.05, ***p < 0.005 versus scramble. Three independent experiments.

Cell intrinsic activation of SHH in *SUFU KD* hNPCs led us to investigate the potential of these modified hNPCs in regulating axonal projections/outgrowth, synapse formation, motoneuronal maturation, and oligodendrocytes differentiation, especially under the condition lacking supplementing growth signaling (e.g., EGF, FGF, GDNF, BDNF, and IGF) that is typically deficient in the injured spinal cord. Neurospheres (passage <5) derived from Scramble and *SUFU KDs* groups were cultured without adding neurotrophic factors. After 7 days of culturing, we observed a robust axonal outgrowth, labeled by the neurofilament marker (NF), in *SUFU KDs* cells, which exhibited an increased number of nerve fibers with much longer extension from the core of neurosphere compared to the scramble control (Fig. 2a, b). In addition, a substantial portion of the *SUFU KD* cells expressed ISLET1/2 (*KD1*:43.9% ± 6.3%; *KD2*:48.0±6.9% versus scramble:3.9±1.0%) among the MAP2^+^ pan-neuronal population on 7 days post-differentiation, indicating accelerated motoneuronal differentiation(Fig. 2c, d). At 21 days, more *SUFU KDs* cells expressed mature motoneuronal marker, choline acetyltransferase (ChAT) and HB9, which were associated with increased expression of the presynaptic marker synaptophysin (Syn) (Fig. 2e, f). The scramble exhibited a higher percentage of undifferentiated hNPCs (39.2± 2.6%) compared to *SUFU KDs* groups(*KD1*: 25.6± 1.9%; *KD2*: 24.8± 2.5%)(Supplementary Fig. 2a, b). Despite *SUFU* *KD* hNPCs primarily favouring motoneuronal fate, we observed a subtle increase in the percentage of postmitotic interneurons marked by Lim1/2+, indicating accelerated or enhanced neurogenesis(Supplementary fig. 2a, b). Consistently, qPCR analysis further confirmed significant downregulation of neural genes(*SOX2* and *PAX6)* and upregulation of motoneuronal-associated genes(*ISLET1/2, HB9, ChAT*), ventral gene(*NKX6.1*) and pan-neuronal gene(*TUJ1*) upon *SUFU KD* (Fig. 2g). These results suggest that knockdown *SUFU* in hNPCs enhances cell intrinsic differentiation potency, promoting neurogenesis and maturation bias toward motor neuron fate in the absence of extrinsic neurotrophic factors support.

Next, we evaluated the glial differentiation capacity, including astrocytes and oligodendrocytes in scramble and *SUFU KDs* hNPCs. After 35 days of culturing in glial differentiation media, GFAP^+^ astrocytes were more frequently observed in the Scramble hNPCs group compared to *SUFU KD* groups (Fig. 2h, i). In contrast, the *SUFU KD* group shows a significantly increased cell population expressing oligodendrocyte markers SOX10 and NG2 when compared to scramble (Fig. 2h, i), most likely due to increased OLIG2 progenitors induced by SHH activation (Fig. 1h). Consistently, the scramble had a higher percentage of undifferentiated hNPCs (26.6±2.4%) compared to *SUFU KD* (*KD1*:17.1±3.0%; *KD2*:16.9±1.7%)(Supplementary Fig. 2c, d). Together, these results suggest that inhibition of SUFU in hNPCs promotes the formation of therapeutic cell types for SCI, including motoneurons and oligodendrocytes.

### Non-cell autonomous effects of *SUFU KD* hNPCs on cell survival and differentiation

**Fig. 3:**
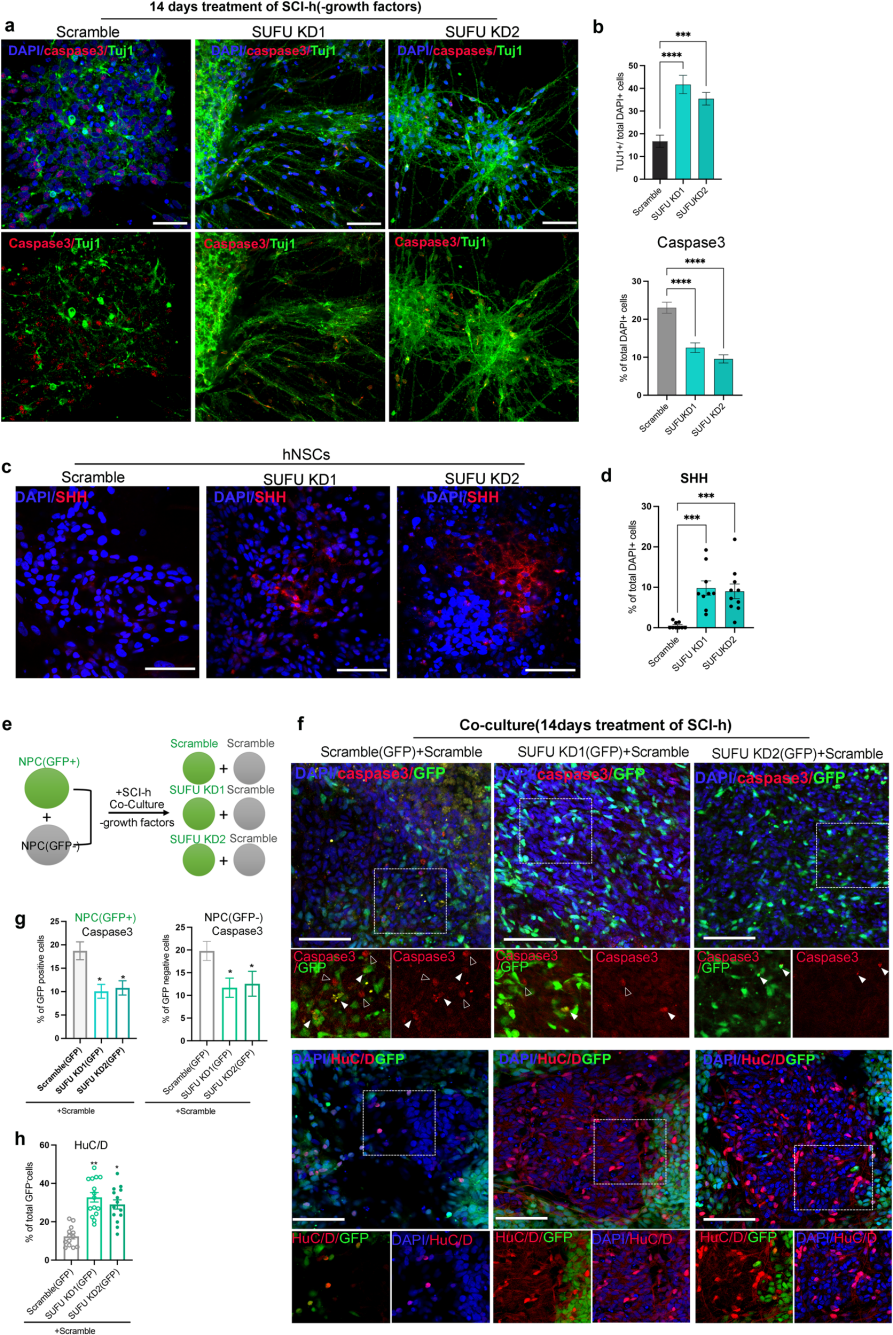
Non-cell autonomous effects of *SUFU KD* cells. **a** Representative immunofluorescence images showing increased SHH production from *SUFU KDs* hNPCs compared to scramble hNPCs. DAPI was used as a nuclei marker, Scale bar = 100 μm **b** Quantification of (**a**). **c** Representative immunofluorescence images of caspase 3, neuronal marker MAP2 and nuclei marker DAPI in Scramble and *SUFU KD* hNPCs after 14 days treatment of homogenate (100 μg/ml) from the injured spinal cord, Scale bar = 100 μm. **d** Quantification of caspase 3+ cells from (**c**). Student’s *t* test. **e** Schematic diagram showing co-culture of scramble and *SUFU KD* hNPCs with and without GFP. **f** Representative immunofluorescence images of caspase 3, HuC/D and GFP in co-cultures of Scramble+scramble(GFP), Scramble+*SUFU KD*1(GFP), and scramble+*SUFU KD*2(GFP), Scale bar = 100 μm. The white box shows a magnified view with indicated markers. **g** Percentage of caspase 3+ cells in GFP-positive or GFP-negative cells in co-cultures of scramble+scramble(GFP), Scramble+*SUFU KD*1(GFP), and scramble+*SUFU KD*2(GFP). One-way ANOVA followed by Tukey’s post-hoc test**. h** Percentage of HuC/D in total GFP cells in co-cultures of scramble+scramble(GFP), Scramble+*SUFU KD*1(GFP), and scramble+*SUFU KD*2(GFP). Student’s *t* test. All data are expressed as mean ± SEM. *p < 0.01, **p < 0.05, ***p < 0. 005. Three independent experiments.

SHH was shown to be neuroprotective and function as a survival-promoting factor for tissue repair. To determine whether *SUFU KD* hNPCs, with intrinsically activated SHH, could counteract the adverse effects from the injured spinal cord niche, hNPCs from scramble and *SUFU KDs* were cultured in the absence of neurotrophic factors and treated with cleared homogenate (100 µg/ml) from the injured spinal cord (SCI-H) for 2 weeks, as described before. Consistent with previous findings^6^, only a small portion of scramble cells differentiated into TUJ1+ neurons(16.7 ± 2.7%) with few axonal outgrowths when exposed to SCI-H. Additionally, we observed a significant increase in apoptosis marked by caspases 3 in scramble cells treated with SCI-H after 2 weeks of culture (Fig. 3a, b). In contrast, *SUFU KD* cells mitigated this effect, generating a substantial amount of neurons(41.7 ± 4.04% in *SUFU KD*1; 35.4 ± 2.8% in *SUFU KD*2) with thick nerve fibers and exhibiting a low percentage of caspases 3 expressions as compared to scramble cells upon treatment of SCI-H(Fig. 3a, b). Notably, the superior neurogenic effects observed in *SUFUKD* hNPCs under an aversive environment cannot be fully achieved through external Shh administration, despite a subtle increase in neuronal populations observed in the presence of SCI-H with SHH supplementation(Supplementary Fig. 2e, f). It is interesting to note that *SUFU KD* cells not only activated SHH signaling intrinsically but also increased SHH protein production in culture, implicating the non-cell autonomous effects via SHH exposure(Fig. 3c, d). To examine whether *SUFU KD* hNPCs could modulate other cell activities, we performed co-culture of *SUFU KDs*(GFP labeling) and scramble(GFP or Non-GFP labeling) (Fig. 3e). Non-GFP scramble NPCs co-cultured with GFP scramble hNPCs or *SUFU KDs* GFP hNPCs were treated with cleared homogenate (100 µg/ml) without supplementing neurotrophic factors. In accordance with the above findings, non-GFP scramble cells cultured with GFP-scramble cells exhibited increased apoptosis with few differentiating neurons(HuC/D^+^) after 14 days of culturing(Fig. 3f–h). Conversely, scramble(non-GFP) cells cultured with *SUFU KDs*(GFP^+^) hNPCs exhibited a significantly lower percentage of apoptosis and were able to form more HuC/D+ neurons after 14 days culturing(Fig. 3f–h). All these data demonstrated the non-cell autonomous effects of *SUFU KD* hNPCs in regulating cell survival and differentiation.

### *SUFU KD* hNPCs modulated the injured niche for survival and connection

**Fig. 4:**
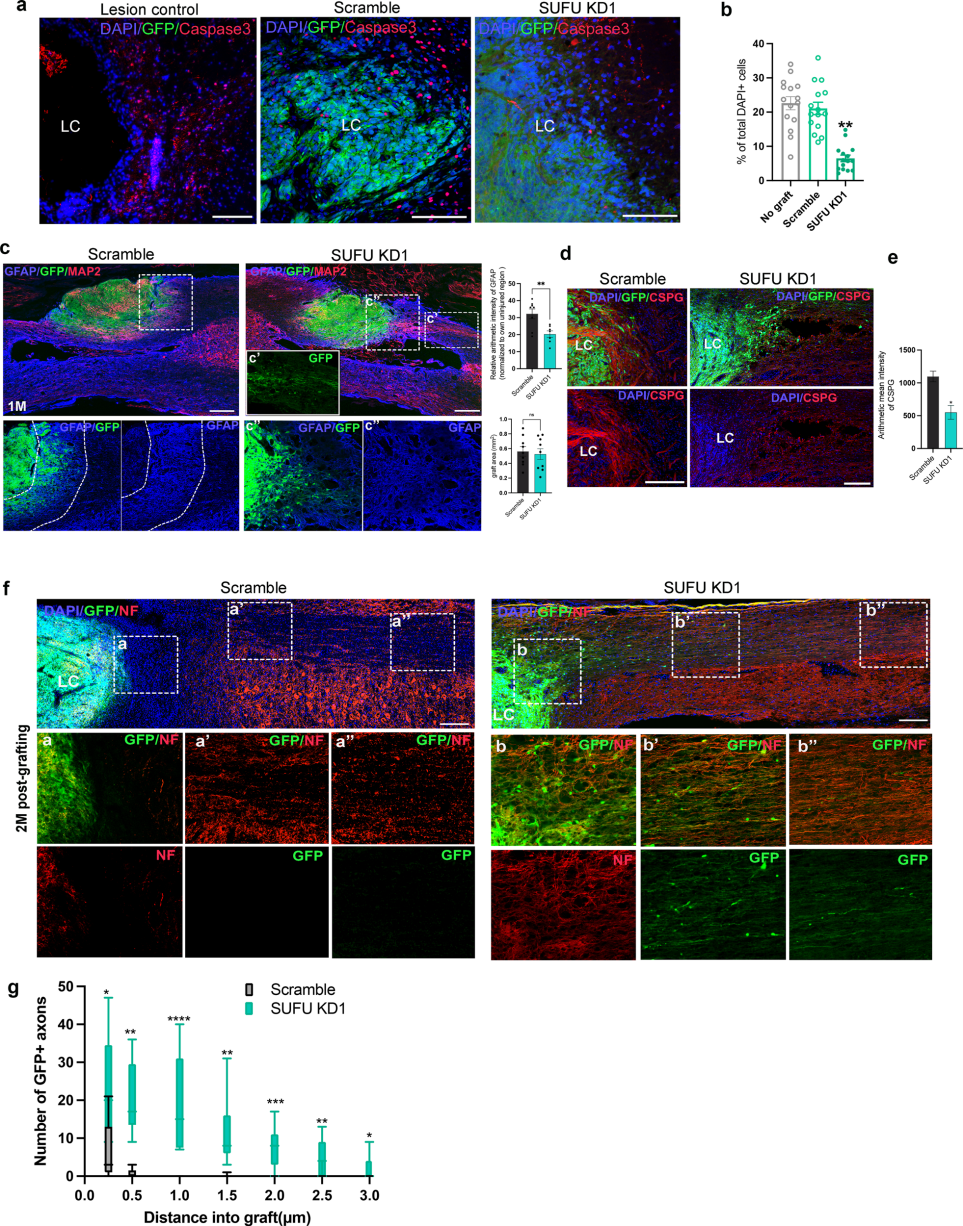
*SUFU KD* grafts display efficient integration and modulate injured niche in the SCI model. **a** Representative images of caspase 3 positive cells in the injured spinal cord without grafting or with scramble and *SUFU KD1* grafts at 1 month(1 M) post-graft, Scale bar = 200 µm. **b** Quantification of the caspase 3 positive cells from (a). One-way ANOVA. *p < 0.05, **p < 0.01. **c** Representative immunofluorescence images of GFP, MAP2 (red), and GFAP (Blue) in sagittal sections with scramble and *SUFU KD1* grafts at 1 month(1 M) post-graft. The cystic lesion cavity (LC) formed with surrounding dense GFAP immunoreactivity (blue), whereas *SUFU KD1* grafts (GFP-positive) crossed GFAP barriers. The white box shows a magnified view with indicated markers in lower panels. Scale bar = 200 µm. The dotted line indicates the dense astrocytic glia marked by GFAP. **d** Representative immunofluorescence images of GFP, CSPG (red), and DAPI (Blue) in spinal cord sagittal sections grafted with scramble and *SUFU KD1* hNPCs at 1 month post-graft (1 M). The cystic lesion cavity (LC) formed with surrounding dense CSPG immunoreactivity (red). *SUFU KD1* grafts with GFP expression attenuated CSPG graft/host interface. Scale bar = 200 µm. **e** Fluorescence intensity analysis of CSPG surrounding the lesion cavity (*n* = 4 rats per group). *p < 0.05**. f** GFP and Neurofilament(NF) immunolabeling in spinal cord sagittal sections revealed GFP-expressing *SUFU KD1* grafts at injured sites generated robust axons extending into the host spinal cord caudally after 2 months post-graft(2 M). insets: a-a” and b-b” indicate higher magnification of NF-positive fibers in the graft at different regions from rostral to caudal. Scale bar = 100 µm. **g** Quantification of axon intercepts at specific distances from graft-host border in the injured cord grafted with scramble and *SUFU KD1* hNPCs. **p < 0.01, ***p < 0.001, one-way ANOVA with Bonferroni. All data are expressed as mean ± SEM.

After confirming the superiority of *SUFU KDs* NPCs in vitro, we further examine whether *SUFU KDs* hNPCs can provide beneficial effects for the injured spinal cord. We induced thoracic contusion injury at level T8 in rats, followed by cell transplantation at 2 weeks post-injury. A total of 2 × 10^5^ GFP-expressing scramble control or *SUFU KDs* hNPCs were grafted into the lesion site (1 µL administered into the left and right sides of the injury site) without growth factors supplementation. Anatomical analysis showed that both scramble and *SUFU KD1* hNPCs could expand and survive in grafted animals (Fig. 4a), indicating successful integration of host tissues at the lesion site and compensation for the dramatic tissue loss within the cavity. In both lesion control and scramble group, a substantial amount of caspase 3+ apoptotic cells were found in the periphery of the lesion epicenter or within the grafts, whereas *SUFU KDs* grafts significantly reduced caspases 3 expressions around lesion sites at 1 M post-grafting(Fig. 4a, b). Consistent with previous findings, the lesion site in the injured spinal cord is surrounded by a glial scar with robust expressions of GFAP, prohibiting nerve extension and reconnection from scramble grafts. Remarkably, the density of GFAP+ glial barrier around lesion sites significantly reduced in the injured spinal cord grafted with *SUFUKD* NPCs. These grafted cells exhibited massive axonal branches, penetrating the GFAP-expressing glial scar as early as 1-month post-graft(1 M)(Fig. 4c). Notably, there were no significant differences of the graft area within the lesion sites between scramble and *SUFU KD1* graft (Fig. 4c).

To determine whether *SUFU KD* grafts facilitate nerve outgrowth by modulating glial scar, we further examined the expression of CSPGs in the injured spinal cord. CSPGs are produced by reactive astrocytes and act as a key inhibitory component to limit axonal outgrowth and regeneration, oligodendrocyte replacement, and remyelination^22^. In agreement with the literature^1,22^, upregulated CSPG expression formed a complex barrier around the lesion sites in response to the injury, restricting the nerve regeneration and outgrowth of scramble grafts (Fig. 4d, e). In contrast, *SUFU KD1* graft significantly inhibited CSPG deposition, which resulted in more dispersed CSPG expressions around lesion sites, leading to a number of GFP+ axonal outgrowth from the grafts for reconstituting neuronal connections (Fig. 4d, e). At 2 months post-grafting, we detected robustly projected neurofilaments(NF+) derived from *SUFU KD1* graft, whereas scramble grafts were restricted within the lesion sites without obvious axonal outgrowth across the lesion site (Fig. 4f). Importantly, a large number of GFP^+^ axons co-expressing NF emerged from *SUFU KD1* grafts into the injured spinal cord and extended much further caudally by more than 25 mm at 2 months post-graft (Fig. 4f, g), recapitulating the long nerve fibers extension observed in vitro. These findings indicate the transplanted *SUFU KD1* hNPCs exert non-cell autonomous effects, which could modulate the injured niche to enhance the survival and the reconstitution of neuronal connections.

### *SUFU KD* grafts promote robust neurogenesis intrinsically and extrinsically in the injured spinal cord

**Fig. 5:**
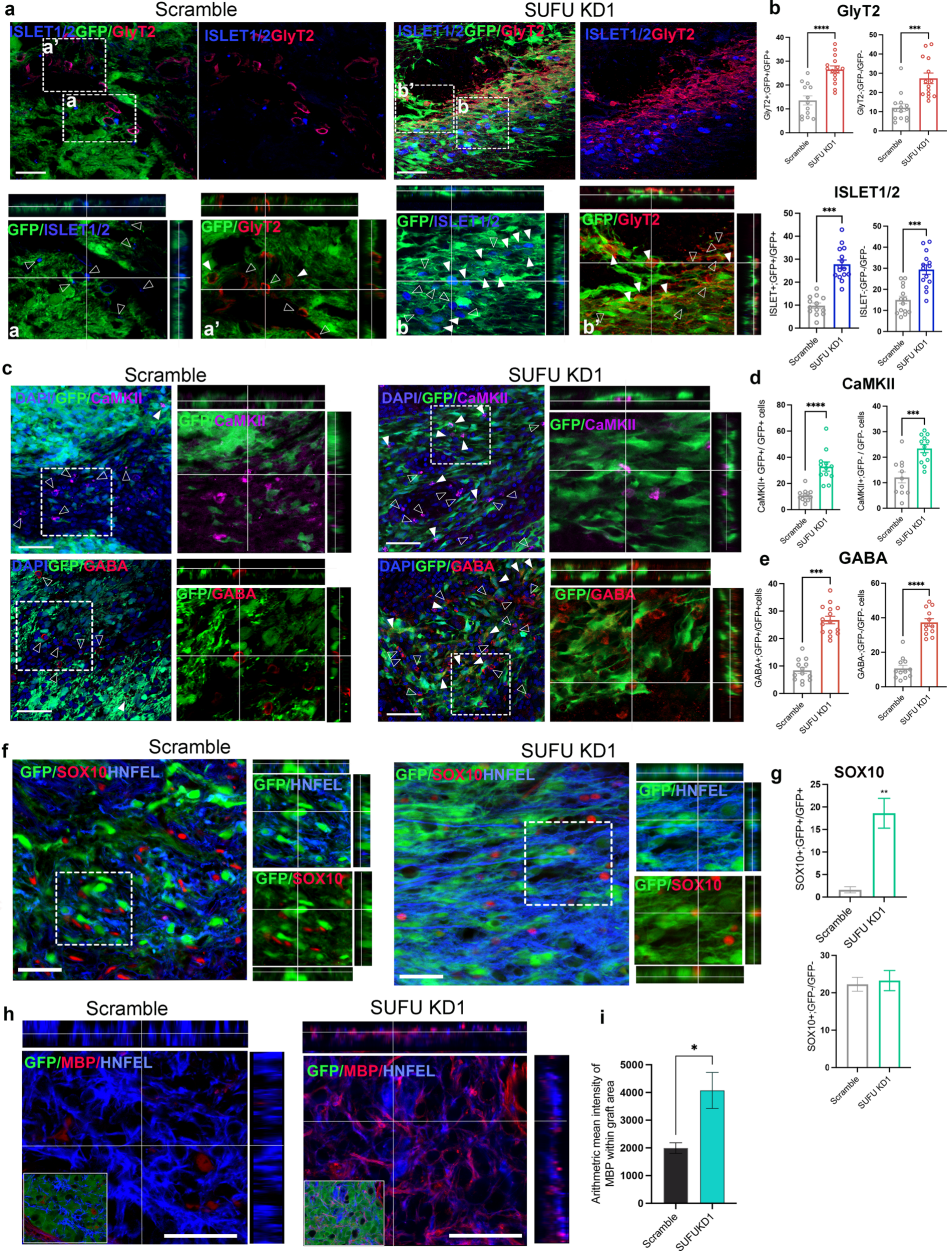
*SUFU KD* hNPC grafts promote beneficial differentiation and regneration intrinsically and extrinsically in the SCI model. **a** Representative immunofluorescence images of GFP, GlyT2(red), and ISLET1/2(blue) in sagittal sections of injured spinal cord with scramble and *SUFU KD1* grafts at 2 month(1 M) post-graft. The empty arrow shows the indicated markers expression in GFP- positive cells. The white arrow shows the indicated markers in GFP negative cells. The white box shows a zoomed-in view of the co-localization of indicated markers. Scale bar = 100 µm. **b** Quantification of the percentage of GlyT2 and ISLET1/2 in grafts or non-grafts cells from(a). **c** Representative immunofluorescence images of GFP, GABA(red) and CaMKII (purple) in sagittal sections with scramble and *SUFU KD1* grafts at 2 month(1 M) post-graft. The empty arrow shows the indicated markers expression in GFP positive cells. The white arrow shows the indicated markers in GFP negative cells. The white box shows a zoomed-in view of the co-localization of indicated markers. Scale bar = 50 µm. *n* = 4–5 rats per group. *p < 0.05, **p < 0.01, ***p < 0.005. Quantification of the percentage of CaMKII (**d**) and GABA (**e**) in grafts or non**-**grafts cells from(c). *n* = 5–6 rats per group. *p < 0.05, **p < 0.01, ***p < 0.005. (**f**) Representative immunofluorescence images of GFP, SOX10(red) and HNFEL(blue) in sagittal sections with scramble and *SUFU KD1* grafts at 2 month(1 M) post-graft. The white box shows a zoomed-in view of the co-localization of indicated markers. Scale bar = 50 µm. (**g**) Quantification of the percentage of SOX10 in GFP-positive or -negative cells in the injured cord grafted with scramble and *SUFU KD1* hNPCs. *n* = 5–6 rats per group, 4–5 sections/rats. Scale bar = 50 µm, **p < 0.01. (**h**) GFP-positive grafts from scramble and *SUFU KD1* immunolabeled with HNEFL and myelination marker (MBP, red) at 3 months post-graft. Insets show the distribution of GFP-positive grafts in the injury site. Scale bar = 50 µm. All data are presented as mean ± SEM.

To further determine whether grafted *SUFU KD1* hNPCs can surmount the injury environment that lacks growth factors and generate therapeutic cell types, we examined the differentiation capacity of scramble and *SUFU KD1* hNPCs grafts. In line with previous findings^3,5,23^, scramble grafts gave rise to less mature neurons while *SUFU KD1* grafts generate a much higher proportion of mature neurons, including motoneurons marked by ISL1/2(*SUFU KD1:* 27.8% ± 4.1% versus scramble: 9.8% ± 4.1%) and ChAT((*SUFU KD1:* 17.9% ± 2.1% versus scramble: 4.1% ± 0.7%), glycinergic inhibitory neurons(*SUFU KD1:* 26.5% ± 5.7% versus scramble: 13.5± 6.9%), CaMKII^+^ excitatory neurons (*SUFU KD1:* 32.9± 12.0% versus scramble: 10.9% ± 4.5%), and GABA^+^ neurons (*SUFU KD1:* 26.8% ± 5.26% versus scramble:8.4 ± 3.9%)(Fig. 5a–e, Supplementary Fig. 3a, b). The total number of GFP cells expressing glutaminergic marker, vGLUT1 was comparable between scramble and *SUFU KD1* grafts. However, *SUFU KD1* cells showed a higher density of vGLUT1 puncta(Supplementary Fig. 3c, d).

Strikingly, we observed significantly increased non-GFP host cells around *SUFU KD1* grafts expressing mature neuronal markers compared to those around scramble grafts, including glycinergic inhibitory neurons (*SUFU KD1*:27.4% ± 10.2% versus Scramble:12.1 ± 7.3%), CaMKII^+^ excitatory neurons (*SUFU KD1*:23.4 ± 5.5% versus scramble12.10% ± 7.01%), motoneurons(ISL1/2, *SUFUKD1:* 29.3% ± 2.3% versus scramble: 15.0% ± 1.9%; ChAT, *SUFU KD1:* 10.6% ± 1.4% versus scramble: 1.1% ± 0.4%) and GABA^+^ neurons (*SUFU KD*:37.3 ± 7.5% versus scramble:10.6% ± 6.3%) (Fig. 5a–e). All these data suggest that *SUFU KD1* grafts not only exhibit enhanced neurogenic potential intrinsically but also exert beneficial effects on the injured environment by promoting neurogenesis of host cells. In a study by Erceg et al.^8^, transplantation of both motoneurons and oligodendrocytes into the SCI of rat models led to better locomotor recovery than transplantation of individual cell types alone. To further investigate whether SUFU grafts also promote oligodendrocytes formation in the injured spinal cord, we examined markers of SOX10 and myelin basic protein (MBP) for oligodendrocytes in the grafts. We found that a substantial amount of SOX10 expression can be detected in *SUFU KD1* grafts, whereas SOX10 expression was barely detectable in the scramble grafts(*SUFU KD1:*18.6 ± 4.2% versus scramble:1.6% ± 1.8%)(Fig. 5f, g). The percentage of non-GFP host cells expressing SOX10 was comparable in both groups, suggesting SUFU grafts did not exert non-cell autonomous effects on regulating oligodendrocyte properties in host. Consequently, *SUFU* *KD1 hNPCs-*derived axons were myelinated, as evidenced by the co-localization of MBP-labeled myelin sheath with HNEFL^+^ axons, whereas no MBP expression was detected with axons emerging from the scramble grafts (Fig. 5h, i). It’s worth noting that the absence of myelination in human axonal fibers in rodent/primate hosts has been reported^24^, which may be attributed to the lack of inter-species recognition. Altogether, these results demonstrate the intrinsic and extrinsic superiority of *SUFU KD* hNPCs in generating therapeutic cell types and modulating injury niche for the treatment of SCI.

### *SUFU KD* grafts effectively establish the integration into host neural circuits

**Fig. 6:**
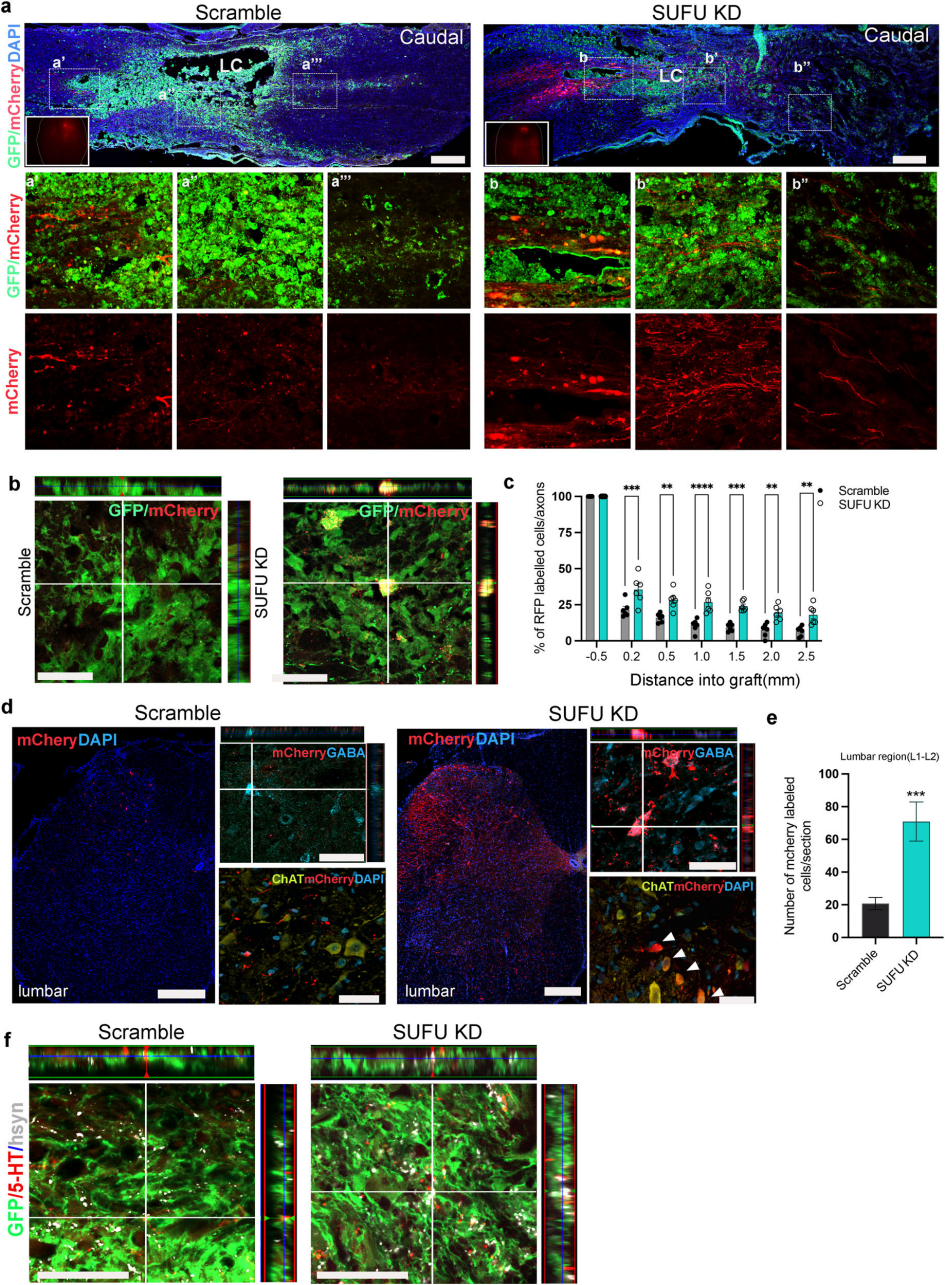
Graft-initiated trans-synaptic AAV virus antegrade labeling of host connectivity. **a** Sagittal section showing antegrade, trans-synaptically traced host mCherry-expressing cells in the injured spinal cord with Scramble and *SUFU KD1* graft. Scale bar=500 μm. Inset, image showing injection sites in the brain region. Inset (a’-a’’’ and b’-b’’’), high-magnification view of boxed area. **b** Representative immunofluorescence images showing mCherry+;GFP+ trans-synaptically connection from motor cortex to the grafts in the lesion sites. Scale bar=50 μm. **c** Quantification of the proportion of mCherry-labeled cells/axons in scramble of *SUFU KD1* grafts, normalized to the total number of mCherry-labeled axons located 0.5 mm rostral to the lesion site. *n* = 7 Scramble recipients and *n* = 6 *SUFU KD1* recipients. One-way ANOVA with Tukey’s multiple comparisons; **p < 0.01, ***p < 0.001, ****p < 0.0001 compared with Scramble grafts**. d** Transverse sections labeled for mCherry and GFP at L2 host spinal cord levels, showing that host neurons monosynaptically connected to grafts were detected over long lengths of the rat spinal cord. Scale bars, 250 μm. left panel showing synaptically connected mCherry+ host neurons included GABA sensory interneurons and ChAT+ motor neurons at the lumbar level. Scale bars=50 μm. The white arrowheads indicate mCherry traced motoneurons(ChAT+) in *SUFU KD1* grafts. **e** Quantification of mCherry traced neurons in L1-L2 in scramble and *SUFU KD1* grafts. *n* = 7 Scramble recipients and *n* = 6 *SUFU KD1* recipients. ***p < 0.001. **f** Triple labeling for GFP, 5-HT, and human synapsin(hSYN) revealed colocalization of regenerating 5-HT axon terminals with hSYN, suggesting synaptic connectivity. Scale bar=25μm. All data are presented as mean ± SEM.

The effectiveness of grafts in integrating with the host neural circuitry and promoting tissue repair for locomotion recovery was evaluated by injecting rAAV-hSyn-CRE + AAV-DIO-mCherry into the motor cortex for antegrade tracing of trans-synaptic connectivity after injury^25^. Histological analysis showed a strong mCherry+ signal in the brain injection sites and injury sites rostrally in the lesion control, scramble and *SUFU KD1* graft groups at 2 weeks post-injections(Fig. 6a and Supplementary Fig. 2). In the lesion control group, the trans-synaptic transmission was severely blocked by the cavity in the injured spinal cord, resulting in absence of mCherry+ cells in the caudal region of the lesion sites, which demonstrated disrupted neural circuits following contusive SCI (Supplementary Fig. 4). In the scramble hNPCs group, only a few mCherry+ cells were observed in the lesion sites and caudal to the lesion site, indicating less effective trans-synaptic transmission. In contrast, strong mCherry signals were detected in the lesion sites overlapping with GFP^+^ cells and several millimeters caudal to the lesion site with *SUFU KD1* graft, confirming trans-synaptic spread of mCherry from host neurons to the graft (Fig. 6a–c). In the lumbar spinal cord, a larger number of mCherry+ cells were present in ventral and dorsal horns of the spinal cord grafted with *SUFU KD1* hNPCs, which co-localized with ChAT+ motoneurons and GABA+ sensory interneurons, probably representing long descending propriospinal tracts in the spinal cord(Fig. 6d, e). These findings indicate that *SUFU KD1* grafts can effectively integrate with both long-projecting and local spinal cord circuitries. In addition, some 5-HT-positive nerve fibers grew into the injury sites and formed synaptic contacts with *SUFU KD1* grafts, whereas few synaptic connections were established between 5-HT-positive nerve fibers and in the area caudal lesion site of scramble graft (Fig. 6f). These results demonstrate that *SUFU KD1* graft effectively promotes the re-establishment of synaptic connectivity with the major host neural circuitry that normally projects to the spinal cord.

### *SUFU KD* grafts improve hindlimb function after contusive SCI

**Fig. 7:**
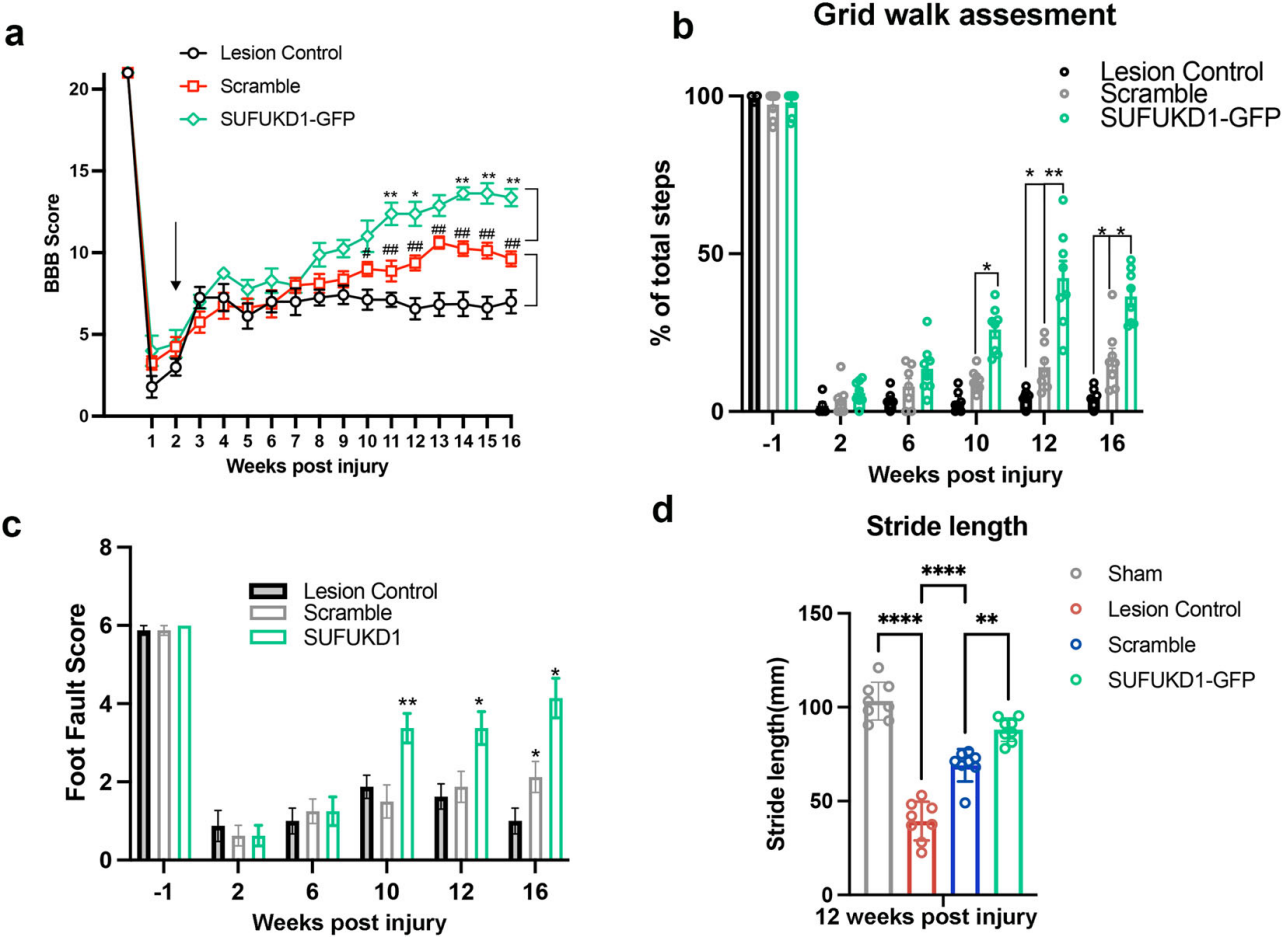
Significant functional improvement after transplantation of SUFU*KD1* hNPC grafts into contusive SCI. **a** BBB scores of lesion control, and pre-and post-grafting with scramble and *SUFU KD1* hNPCs. Two-way repeated-measures ANOVA followed by post-hoc Fisher’s exact test. *p < 0.05, **p < 0.01 *SUFU KD1* versus scramble; ^#^*p* < 0.05, ^##^*p* < 0.01 scramble versus lesion control. **b** Grid walk quantitative analysis measured as a percentage of hind limb placement. One-way ANOVA with Tukey’s post-hoc test; *p < 0.05, **p < 0.01. **c** Foot fault score analysis of hind limb measured by rating scale for foot placement in the skilled ladder rung walking test (correct placement = 6 points; partial placement = 5 points; placement correction = 4 points; replacement = 3 points; slight slip = 2 points; deep slip = 1 point; and total miss = 0 points). One-way ANOVA with Tukey’s post-hoc test; *p < 0.05, **p < 0.01. **d** Quantification of stride length in sham, SCI(lesion control) and SCI rats with scramble and *SUFU KD1* grafts. Student’s *t* test. *p < 0.05, **p < 0.01. All data are expressed as mean ± *S*EM. *n* = 7(sScramble); *n* = 5(lesion Control); *n* = 9(*SUFU KD1)*; *n* = 6 (Sham).

To evaluate the effect of scramble and *SUFU KD* grafts on functional recovery after SCI, we performed a series of motor function tests during the 16-week post-injury period. Hindlimb locomotor activity in the lesion control, receipts with scramble and *SUFU KD* grafts was assessed weekly by the Basso, Beattie, and Bresnahan (BBB) locomotor scale, starting 7 days before and after injury. At 1 and 2 weeks post-injury, all treatment groups experienced a dramatic loss of locomotor function, which is attributed to the “spinal shock” associated with the transient shutdown of sensorimotor function in the spinal cord acutely after SCI^26^. From 3 to 4 weeks onwards, the BBB score of all groups reached a relatively stabilized level(around 7) during the subacute and chronic phases of injury, showing the similar level of locomotion activity reported previously^27–29^. Scramble recipients started to show significantly improved locomotor function from 12 weeks (10 weeks post-graft) compared to lesion control(^#^*p* < 0.05, ^##^*p* < 0.01), leading to an increase in BBB score from 7(extensive movement of all three joints of the HL) to 11, which aligns with the effects reported in other studies using unmodified NPCs^30,31^. Notably, *SUFU KD* recipients demonstrated quicker improvement in hindlimb motor function starting from 10 weeks (8 weeks post-graft) until the end of the experiment (16 weeks post-injury), as compared to lesion control (Fig. 7a). In addition, *SUFU KD1* recipients showed much greater improvement in hindlimb motor function compared to scramble recipients animals from 11 weeks post-injury (*p < 0.05,**p < 0.01) (Fig. 7a), resulting in a significant recovery of the BBB score from approximately 7 to 13. To further evaluate skilled locomotor function and coordination, the grid-walking test was employed by counting the percentage of correct steps out of paw replacements and foot faults as rats traversed the metal grid^32^. After injury, rats lost the ability to place their hind paw correctly on the metal grid. Starting from 10 weeks post-injury, the *SUFU KD1* grafted rats exhibited much better performance in placing their affected hind paw (left and right) correctly on the grid with less misdirected steps compared to the scramble recipients, which showed a mild improvement by week 12 compared to lesion control(Fig. 7b, c). Subsequently, the gait of the transplanted animals was assessed by the footprint test, in which the pressure exerted by the feet during locomotor activity was converted into a digital image of the plantar surface by a force sensor, indicating limb stepping ability and coordination. Quantitative analysis of stride length in grafted animals further confirmed a marked improvement in *SUFU KD* grafted animals with values higher than those of scramble and lesion controls (Fig. 7d). These findings demonstrate the enhanced therapeutic potential of *SUFU KD1* grafts for the restoration of locomotor function in a rodent model of contusion SCI.

## Discussion

Traumatic spinal cord injury, caused by compression, contusion or laceration, often leads to rapid necrotic death and loss of neurons and glia. This is followed by the activation of astrocytes and inflammatory responses, resulting in widespread changes in extracellular matrix molecules in the microenvironment. Consequently, the complex post-injury milieu and the failure of nerve tissue regeneration following SCI causes varying degrees of paralysis, depending on the level and severity of the injury^1,28^. Stem cell transplantation, especially hNPCs derived from hPSCs, offers a potential strategy for the treatment of SCI. These grafts have been shown to be a promising cell source for repairing injured spinal cord because of their competency to generate neurons for restoration of neuronal circuitry and oligodendrocytes for remyelination of spared axons. However, the functional outcome of grafted hNPCs that largely depends on the survival, differentiation potency, integration capacity and axial identity is hampered by the hostile micro-environment at and around the lesion sites^4,33,34^. Additionally, the amount of oligodendrocytes generated is often insufficient for remyelination of spared axons^2,3,24^. Previous studies have shown that activation of signaling pathway, such as SHH and GDNF, in the post-injury microenvironment can modulate the niche and enhance the viability and differentiation potency of the grafted cells. Nevertheless, the beneficial effects of these growth factors on the grafted cells and the niche might be transient and limited by the prolonged differentiation and maturation process of human cells^6,12,13^. In this study, we demonstrated that hNPCs with SUFU inhibition activated SHH signaling, promoting survival and intrinsic differentiation in generating therapeutic cell types without reliance on trophic factors support. Remarkable, these modified hNPCs also exert non-cell autonomous effects, which facilitate endogenous neurogenesis and modulate microenvironment by reducing progressive apoptosis and CSPG+ suppressive barriers around lesion sites. The combined beneficial effects of SUFU inhibition in hNPCs greatly improved locomotor functions in a severe traumatic injury model.

Sonic Hedgehog (Shh) is a secreted glycoprotein that plays a crucial role in neurodevelopment through its ability to regulate renewal and survival of neural progenitors, differentiation, axon growth and cell fate determination of motoneurons and oligodendrocytes^7,8^. The superiority of SHH activation intrinsically and extrinsically has been shown in various perspectives for the treatment of SCI. For example, the activation of SHH by small molecules(e.g., purmorphamine) in vitro is necessary to expand hNPCs populations, and generate motor neurons and oligodendrocytes for compensating the cell loss in the injured spinal cord and promoting locomotion recovery after transplantation^35^. SHH can exert neuroprotective effects on the microenvironment of CNS injuries by increasing the proliferation of endogenous NG2+ oligodendrocyte lineage cells, reducing astrocytic scar formation, and limiting inflammatory responses. In addition, long-term administration of SHH recombinant protein facilitated the endogenous axonal sprouting and growth^9,36–38^. During vertebrate development, SUFU plays a critical role as a negative modulator of SHH signaling pathway. Ablation of *Sufu* results in excess Shh production in the neural tube^39^. Notably, SUFU exhibits a distinct mode of action compared to other SHH negative effectors(e.g., Patch 1), which does not cause tumorigenic upon the deletion in vivo, providing a strong rationale for performing *SUFU* knockdown in hNPCs rather than other SHH effectors for transplantation therapy in SCI rodent model^40^. Here, we genetically inhibited *SUFU* expression in hNPCs to activate SHH signaling pathway. This approach enabled neural stem/ progenitor cells or injured tissue to aid SCI repair, exerting strong therapeutic potential both cell and non-cell autonomously. Despite a previous study indicating that Shh administration could not increase the NPC-differentiation toward the neuronal lineage under LPS-induced pro-inflammatory conditions^12^, our finding revealed an increased neuronal population upon Shh supplementation in the presence of SCI-H. This discrepancy may be attributed to variations in NPC sources and species, as well as the injury niche. Tail et al. utilized NPCs isolated from the postnatal rat brain region, while our study employed NPCs derived from hPSCs, potentially resulting in differential neurogenic capacity and responses to the external stimuli. Furthermore, the inflammatory responses induced by LPS differ from the complex disruptive signaling molecules associated with the injured environment from SCI-H. Importantly, the superior neurogenic effects observed in *SUFUKD* hNPCs under an aversive environment cannot be fully achieved through external Shh administration, implying reduced SUFU in hNPCs may also involve the activation/repression of other signaling pathway that potentially exert beneficial effects on survival and differentiation.

The functional recovery achieved by hNPCs graft is largely determined by survival, cell replacement and integration into the host tissue. It has been known that the injured microenvironment in adults is astrocytic and lack of neurotrophic factors, which favors GFAP+ astrocytes induction and restricts neurogenesis of transplanted hNPCs. Compared to scramble grafts, SUFU*KD* grafts were more resilient to the detrimental environment after SCI, showing better survival and strong neurogenic potency in generating substantial amounts of mature neuronal subtypes compared to scramble grafts in the absence of trophic support. These observations are in line with previous findings that repression of SUFU in progenitors from different regions of the CNS or PNS is required for the onset of neuronal or glial differentiation^16–18^. In addition, a previous study has revealed that Sufu could be a direct target of Sox10 and Sox10 directs mouse NPCs toward the oligodendrocyte lineage by decreasing Sufu expressions^16^. Thus, hNPCs with SUFU inhibition also exhibit a strong ability to generate oligodendrocytes marked by SOX10 and NG2, which facilitated the myelination of axons outgrowth and contributed to the locomotion recovery following the SCI. Notably, matched grafts were able to rewire the lesioned spinal cord^3,33,41^. Accordingly, *SUFU KD* grafts reduced apoptosis and formation of inhibitory astrocytic scar expressing CSPGs around the lesion cavity, which enabled long-distance axonal growth into the caudal recipient spinal cord beyond the lesion site. Interestingly, *SUFU KD* grafts also promoted neuronal differentiation and maturation of non-modified hNPCs and host cells, as we consistently observed a significant increase in subtypes of neuronal markers around *SUFU KD* hNPCs in vitro and in vivo. The non-cell autonomous effects observed from *SUFU KD* hNPCs could be due to the upregulation of SHH ligands, which exert impacts on adjacent cells and the injured niche. The rewired post-injury microenvironment by *SUFU KD* hNPCs appears to be less aversive and astrocytic to endogenous cells, while produced SHH ligands can act as a trophic factor^42,43^ to support non-modified hNPCs and endogenous host cells, thus leading to increased survival, proliferation, and neuronal differentiation. It is also possible that altered SUFU activities may potentially regulate or influence other signaling factors, such as BDNF, WNT or Notch, contributing to the overall changes in the responsiveness of hNPCs and neurons^44–46^.

These findings represent a key advancement in enhancing the therapeutic efficacy of hiPSCs-derived hNPCs transplantation for treating SCI. Our approach alters the grafts’ response in the injury niche, resulting in enhanced differentiation capacity, survival, and integration. Moreover, these modified grafts also rewire the niche and endogenous tissues. To enable the clinical translation of our findings, it is essential to evaluate the therapeutic effects of *SUFU KD* hNPCs in larger mammals, such as primate or pig, as there are numerous differences in the size, anatomy and disease/developmental time scale, and physiology of the spinal cord between rodents and humans. These innovations hold great therapeutic promise and may yield hNPCs with unique cell and non-cell autonomous properties for successfully treating SCI.

## Methods

### Pluripotent stem cell culture

Human pluripotent stem cells (IMR90, H9, and HES2) were provided by WiCell Research Institute (Madison, WI), and passaged 33–49. Cells were cultured on Matrigel gel (Corning)-coated plates in mTeSR (Stem Cell Technologies) or E8 medium (Thermo Fisher Scientific, Waltham, MA). Cells were passaged with ResLR (Stem Cell Technologies), washed, and replated at a dilution of 1:5 or 1:10.

### Medium for neural induction, maintenance, and differentiation

For N2B27 medium, we mixed 5 mL of N2 supplement (100x, serum-free), 10 mL of B27 supplement (serum-free, 50×), 10 mL of MEM non-essential amino acids (100×), 5 mL of penicillin-streptomycin, and 5 mL of Glutamax in DMEM/F12 medium, making up to 500 mL. All reagents were purchased from Thermo Fisher Scientific. To prepare 10 mL of neural induction medium, we mixed 10 mL of N2B27 medium with 250 nM LDN-193189 (Tocris Biosciences, stock in DMSO), 10 μM SB-431542(stock in DMSO), 4 μM CHIR-99021 (Tocris Biosciences, stock in DMSO), and 250 nM RA (Tocris Biosciences) in an aseptic environment. For neural maintenance medium, N2B27 medium was supplemented with 300 ng/mL cAMP (Sigma-Aldrich), 0.2 mM vitamin C (Sigma-Aldrich), 20 ng/mL EGF and 20 ng/mL FGF (Peprotech, Rocky Hill, NJ). For neuronal differentiation medium, N2B27 medium was supplemented with 20 ng/mL of BDNF, 20 ng/mL of GDNF, and 20 ng/mL of IGF(Peprotech, Rocky Hill, NJ). For neural medium used to assay the spontaneous differentiation capacity of NPCs, N2B27 medium was supplemented with 300 ng/mL cAMP (Sigma-Aldrich) and 0.2 mM vitamin C (Sigma-Aldrich) without any other trophic factors. For glial differentiation, N2 medium was supplemented with 10% FBS and 30 ng/mL T3.

### Neural induction, maintenance, and neurosphere culture

To induce PAX6- and SOX2-expressing neuroepithelia (NE) formation, human pluripotent stem cells were suspended on a low-attachment plate for 2 days in neural induction medium and then plated on Matrigel-coated plates, as previously described^47^. The medium was replaced with fresh neural induction media every 2 days. On days 7–9, neuroepithelial cells formed neural tube-like rosettes, which were gently selected using a 200 μL pipette on day 10, and cultured with neural maintenance medium in Matrigel-coated wells or low attachment plate. An addition of 10 μM Y-27632 (ROCK inhibitor; Tocris Biosciences) was applied to enhance cell survival at each passage.

For the neurosphere assay, hNPCs at passage 1 were dissociated into a single-cell suspension at 10^5^/mL and transferred onto 6-well low attachment plates (Corning). Neurospheres were cultured in neural maintenance medium, with 3 mL added in each well. For secondary neurosphere formation, primary neurospheres were further dissociated into a single-cell suspension after 7 days, and 10^5^/mL of the cell suspension was transferred into 6-well low attachment plates for further culture.

### Constructs and cell lines

We designed shRNA against human *SUFU KD*1(5′AACAGAGTCCATGAGTTTACA3′) and *SUFUKD2(5*′AACAGTGAGTCAAGAATTCAG3′) and using the principles from The RNAi Consortium (https://www.broadinstitute.org/rnai/public /), and then cloned them into lentiviral vector pLKO.1-puro/eGFP and the pLKO.1-TRC control (Addgene plasmid #10879). The human *SUFU* cDNA was cloned into lentiviral vector pLVX-EF1α-puro (Clontech). To produce lentivirus, production, 5 × 10^6^ 293T cells were plated in a 100-mm dish and transfected with a lentiviral expression vector, packaging plasmid psPAX.2, and envelope plasmid pMD2.G using PolyJet™ (SignaGen). The cell culture medium containing the lentiviral particles was harvested at 48 and 72 h post-transfection, and filtered through a 0.45-μm filter. The lentiviral particles were titrated by Lenti-X qRT-PCR Titration Kit(Takara). Next, 3 × 10^5^ human pluripotent stem cells were infected with quantified lentivirus particles expressing cDNA and/or shRNA and cultured in the presence of 8 μg/mL Polybrene (Sigma) for 24 h. After 48 h of transduction, infected cells were screened in the presence of 1 μg/mL puromycin (Life Technologies). For enrichment of GFP-expressing cells for transplantation, GFP-positive hiPSCs or NPCs derived from GFP-hiPSCs were expanded after flow cytometry(BD FACSMelody, Supplementary fig. 5b). Briefly, the cell suspension was washed in DMEM and filtered through a 50-μm nylon cell strainer. The single-cell suspension was collected by centrifugation and suspended in mTesR or neural maintenance media and then subjected to Fluorescence-activated cell sorting (FACS). Enriched GFP-positive cells were expanded for 3–4 weeks (up to five passages) before subsequent analysis and experiments.

### Immunocytochemistry

Cultures were fixed for 30 min in 4% paraformaldehyde (PFA) in 0.1 M phosphate buffer at 4 °C. After washing three times with PBS, fixed samples were permeabilized with 0.1% Triton X-100 with 1% BSA in PBS for 1 h at room temperature. Primary antibodies in blocking solution were added and incubated overnight at 4 °C(Supplementary Table 1). After rinsing, samples were incubated with donkey Alexa Fluor–conjugated secondary antibodies (1:500; Invitrogen) for 1 h at room temperature. Nuclear counterstaining was carried out with 4′,6-diamidino-2-phenylindole (DAPI). Images were captured by a confocal microscope (LSM 800 or 900, Zeiss) using 20× or 40× (oil) objective. Marker-positive and total cells (DAPI or GFP) were quantified by ImageJ software or photoshop combined with manual counting for different types of cells, including neural progenitor, neurons, astrocytes and oligodendrocytes. four to six images from left to right, and then up and down were captured at 200× or 400× magnification.

### Quantitative reverse-transcription polymerase chain reaction

Total RNA was isolated from the culture using the RNeasy mini kit (Qiagen, Hilden, Germany) according to the manufacturer’s protocol. Total RNA was quantified with NanoDrop (Thermo Fisher). For cDNA synthesis, the reverse-transcription reaction was carried out with PrimeScript RT master mix (Perfect Real Time, Clontech), and quantitative PCR was performed using primers specific for the genes of interest (Supplementary Table 2) with SYBR Premix Ex Taq II (Tli Rnase H Plus; Clontech) in 20 μL reaction volumes. We used the ΔΔCt method to calculate the relative gene expression with Actin as the internal control.

### Western blotting

Cells were washed twice in cold phosphate-buffered saline (PBS) and lysed in RIPA buffer (150 mM NaCl, 1 mM EDTA, 1% NP40, 0.5% Sodium deoxycholate, 0.1% SDS, 50 mM Tris-HCl, pH 7.5) supplemented with 1% protease and phosphatase inhibitor cocktail (Thermo Fisher). Proteins were separated by SDS-PAGE using a Bio-Rad system under reducing conditions. Membranes were probed with antibodies against SUFU (cell signaling) and Actin overnight at 4 °C and then incubated with appropriate horseradish peroxidase-conjugated goat anti-rabbit (at 1:2000, Dako) at room temperature for 1 h. After incubation with the ECL substrate for 1–3 min, blots were exposed to X-ray film (FujiFilm Super RX) at different times to obtain the optimal intensity of the protein bands, and images were analyzed by ImageJ.

### Animals

We used Sprague–Dawley male rats (230–260 g) from the Centre for Comparative Medicine Research, Li Ka Shing Faculty of Medicine, The University of Hong Kong. All animal protocols were approved by the Committee on the Use of Live Animals in Teaching & Research of The University of Hong Kong. Health guidelines for laboratory animal care and safety were strictly followed. Animals had free access to food and water throughout the study.

### SCI contusion surgery (Severe)

Sprague–Dawley male rats were anesthetized with an intraperitoneal injection of a ketamine (80 mg/kg) and xylazine (10 mg/kg) mixture. The paravertebral muscles at the region of T6–T12 were removed and a laminectomy was performed at the T8(vertebra), and a T8 severe contusion SCI (weight of 25 g, height of 50 mm) was produced with a modified version of MASCIS impactor^48^. After performing the spinal contusion, muscle and skin layers were sutured with 4.0 polyglactin. The bladder of each injured animal was squeezed manually twice a day after SCI for 2 to 3 weeks.

### Preparation of spinal cord homogenate

The spinal cord homogenate was made with modifications from previously published protocol^6^. In brief, rats were perfused with ice-cold saline two weeks after injury, and around a 5 mm long segment of the injured spinal cord centered on the injury epicenter was collected and rapidly frozen on in liquid nitrogen. Spinal cords from at least 4 SCI rats were pooled together and were grind to a fine powder under liquid nitrogen by using pestles, while kept on ice. The homogenate was suspend in DMEM/F12 and cleared by centrifugation at 12,000*g* for 10 min at 4 °C. The total protein concentration of cleared supernatants was measured using a BCA test. After adjusting the total protein concentration, the aliquots were kept at −80 °C until use.

### Cell engraftment

Fourteen days after SCI surgery, animals underwent a second procedure for cell implantation. The rats were anesthetized with an intraperitoneal injection of a ketamine (80 mg/kg) and xylazine (10 mg/kg) mixture. The original incision was reopened, and the injury sites were re-exposed. To investigate the survival and spontaneous differentiation capacity of scramble and *SUFU KD* hNPCs, Scramble and *SUFU KD* hNPCs were resuspended in DMEM/F12 medium supplemented with 10 µM Rock inhibitor at a density of 10^5^ cells/µL without growth factors. Cells were kept on ice throughout the procedure. Two injections were performed at the injury site, each delivering 2.0 µL of the cell suspension at −0.5 and +0.5 mm distance from the dorsal middle line (0 mm). The injection was performed using a 30 G syringe (Hamilton) connected with a micropump (RWD), with the animals tightly fixed in a stereotaxic apparatus (RWD). The injection rate was 250 nL/min. The syringe was left in place for an additional 10 min before and after the injections. At the end of the procedure, the muscle and skin layers were sutured with 4.0 polyglactin, and the animals received subcutaneous injections of buprenorphine (0.03 mg/kg) and meloxicam (2 mg/kg) for 3 days, and oral administration of enrofloxacin (2.5%) for 7 days. Cyclosporine (5 mg/kg) was subcutaneously injected every day for immune suppression. Animals underwent functional testing for up to 12 weeks and were sacrificed for anatomical analysis by transcardial perfusion with 4% formaldehyde.

### Injection of viruses for anterograde trans-neuronal labeling

Three weeks before sacrifice, the skulls of anesthetized rats (ketamine (80 mg/kg) and xylazine (10 mg/kg) were tightly fixed to a stereotaxic apparatus (RWD). rAAV-hSyn-CRE-WPRE-hGH pA(1 × 10^13^ vg/mL) + AAV-DIO-mcherry (brainVTA, 2 × 10^12^ vg/mL) was injected into five spots of the right motor cortex. A vertical midline incision was made from between the eyes to the posterior skull. The injection area on the right hemisphere defined in a rectangle measuring 2 mm (from 1.0 mm anterior to −1.0 mm posterior to the bregma) by 1.5 mm (lateral to the bregma). A drill was used to create the injection sites on the skull. Injections were performed using a 33 G syringe (Hamilton) attached to a micropump (RWD). Each injection delivered 0.5uL of the virus solution into the motor cortex at a rate of 100 nL/min. The injector tip was left in place for an additional 5 min before and after the injections. Animals were euthanized 3 weeks following injection and postsynaptic structures were examined for the presence of cell body labeling.

### Histology and immunohistochemistry

Animals were euthanized by intraperitoneal injection of pentobarbitone overdose (150–200 mg/kg) before perfusing with 0.9% saline followed by 4% PFA. Spinal cords were removed, post-fixed, and sectioned at 20-μm intervals. Sections were dried overnight at room temperature. Sections were then incubated with primary antibodies overnight (Supplementary Table 1) followed by incubation with Alexa Fluor 488, 594, or 647–conjugated donkey secondary antibodies (1:500; Invitrogen) for 1 h at room temperature. For nuclear staining, DAPI was added to the final wash. Images were captured under confocal microscopy (LSM 800 or 900, Zeiss) using 10×, 20×, or 40×(oil) objective using Z-stack or tiles.

### Quantification of mCherry labeled cells/axons or neural cell type in grafts

For quantification of neural differentiation or growth in human cell grafts, eight to nine randomly selected fields of grafts from six to eight animals per group were visualized using a Carl Zeiss LSM 800 or 900 confocal microscope at a magnification of 100× or 200×. The number of mcherry-expressing axons regenerating into grafts in the lesion sites was quantified as previously described^3,33^. In brief, Using ZEN offline, dorsal-to-ventral virtual lines of one in six sections(30 µm thickness) were placed at regular distances under 100× magnification, and graft/host interface and the number of axons intercepting labeled for 5-HT or mCherry-labeled axons were examined and counted under 200× magnification.

### Quantification of glial scars

Sections were fluorescently immunolabeled for GFAP or CSPG and GFP to quantify the percentage of the field occupied by GFAP immunoreactivity surrounding the graft-lesion cavity or lesion cavity alone, as previously described^49^. A 100-µm wide zone surrounding the lesion site was quantified in this manner.

### Behavioral studies for locomotor activity

A total of 91 rats underwent SCI and were randomized to receive scramble or SUFU *KD* grafts in different batches. The BBB open-field 21-point locomotion rating scale was used in the weekly assessments of rats conducted by two independent observers^50^. Grid walk assessment was performed using a modified grid (4 × 6 cm grids), and hind limb foot drops were recorded as a measure of hind limb sensorimotor function. Two investigators blinded to group identity assessed outcomes. Foot drops were recorded if the rat was unable to grasp a grid rung with a hind paw during stepping and paw placement, resulting in a foot drop below the grid. The percentage of total paw replacements = Total steps–foot drops / Total steps (including left and right hind limb). Testing was performed on uninjured rats prior to surgery as a baseline measurement, and then every 2 to 3 weeks post-injury. Three trials per rat were evaluated and the scores were averaged for the analysis. For the foot fault scoring, a qualitative analysis was performed on skilled walking as previously described^32^. Briefly, the score was defined as follows: correct placement = 6 points; partial placement = 5 points; placement correction = 4 points; replacement = 3 points; slight slip = 2 points; deep slip = 1 point; and total miss = 0 points. With the limb that started the walk, consecutive steps were then estimated. Three trials per rat were evaluated and the scores were averaged for the analysis. For the footprint analysis, animals were placed on a 1-m long narrow corridor lined with force sensors that recorded the pressure exerted by the feet due to locomotor behavior, which was converted into digital image of plantar surface, which reflects the hind limb supporting ability and coordination. Rats were then allowed to run three times along the corridor, and stereotyped gait and motor coordination parameters, including hind limb stride length and width were measured from three complete step cycles from the middle of the runway^23^.

### Statistical analysis

For comparison between two groups two-tailed Student’s *t-test* was used at a designated significance level of *p* < *0.05*. The normality assumption was verified using the Shapiro-Wilk test.

Measurements taken at different time points were compared using one-way repeated-measures ANOVA or two-way repeated-measures ANOVA, followed by Tukey post hoc or Bonferroni test. Gene expression data were analyzed using two-tailed one-sample Student’s *t* tests when compared to baseline control group. Statistical analyses were performed using Prism 8 (Graphpad Software) with a designated significance level of 95%. All data were presented as mean ± SEM. The statistical details of each experiment can be found in the figure legends. No statistical methods were used to calculate sample size estimates.

### Reporting summary

Further information on research design is available in the Nature Research Reporting Summary linked to this article.

### Supplementary information

Supplemental material

Reporting summary

## Data Availability

All data generated or analyzed during this study are included in this article and its supplementary information files. All other data collected and analyzed during the current study are available from the corresponding author upon reasonable request.

## Code availability

Public software and custom code described in the Methods section were used to process the data in this paper.
