# Supplementary material for: Intrinsic and extrinsic actions of human neural progenitors with SUFU inhibition promote tissue repair and functional recovery from severe spinal cord injury
Source: NPJ Regen Med. 2024 Mar 22;9:13. doi: 10.1038/s41536-024-00352-4 (PMC10959923; doi:10.1038/s41536-024-00352-4)
Supplement: Supplementary file 1 — Supplemental material [file 41536_2024_352_MOESM1_ESM.pdf]

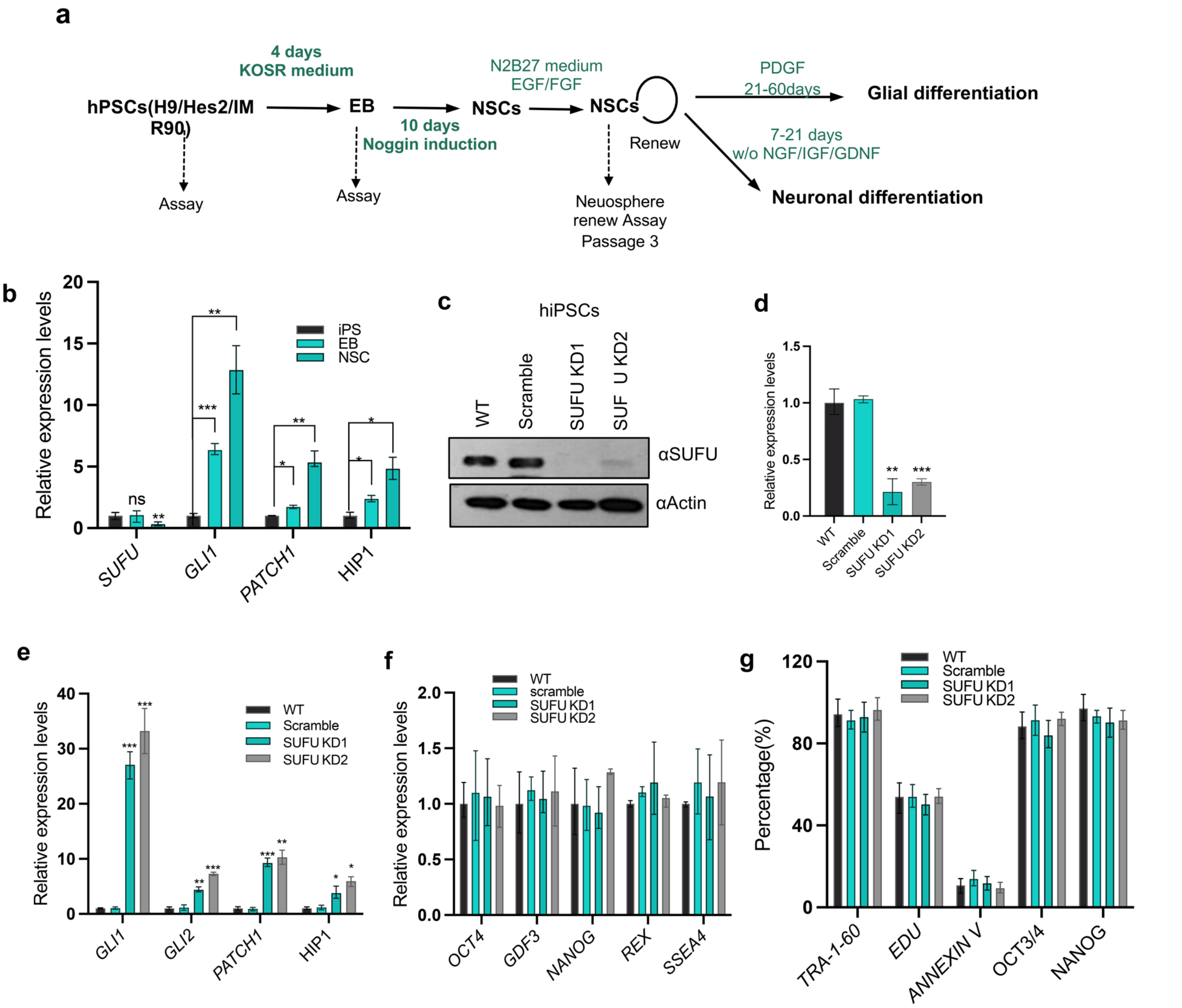

Supplementary Figure 1. Activation of SHH signaling has no effects on hiPSCs **(a)**Schematic showing steps involved in differentiation of human pluripotent stem cells (hPSCs) into human neural stem cells(hNSCs). **(b)**qRT-PCR analysis of indicated hedgehog(HH) effectors in hiPSCs, EBs(7 days) and hNPCs(passage 3). Gene expression was normalized to *36B4* and shown relative to hiPSCs, which is arbitrarily defined as 1. **(c)**WB analysis of SUFU proteins in WT, Scramble, *SUFUKD1* and *SUFUKD2* hiPSCs. **(d)** qRT-PCR analysis of *SUFU* mRNA levels in WT, Scramble, *SUFUKD1,* and *SUFUKD2* hiPSCs. **(e)** qRT-PCR analysis of *GLI1, GLI2, HIP1*, and *PATCH1* mRNA levels in WT, Scramble, *SUFUKD1*, and *SUFUKD2* hiPSCs. **(f)**qRT-PCR analysis of hPSC markers in WT, Scramble, *SUFUKD1*, and *SUFUKD2* hiPSCs. **(g)**FACS analysis of pluripotent markers (TRA-1-60, OCT3/4, NANOG), apoptotic marker (ANNEXIN V) and EdU incorporation assay in WT, Scramble, *SUFUKD1* and *SUFUKD2* hiPSCs. The expression values are shown in percentages of total cells within the gate. Significant change is compared to WT. For all the experiments, the expression values are mean ± SE of three independent experiments. * $p < 0.05$; ** $p < 0.01$, ***$p<0.001$.

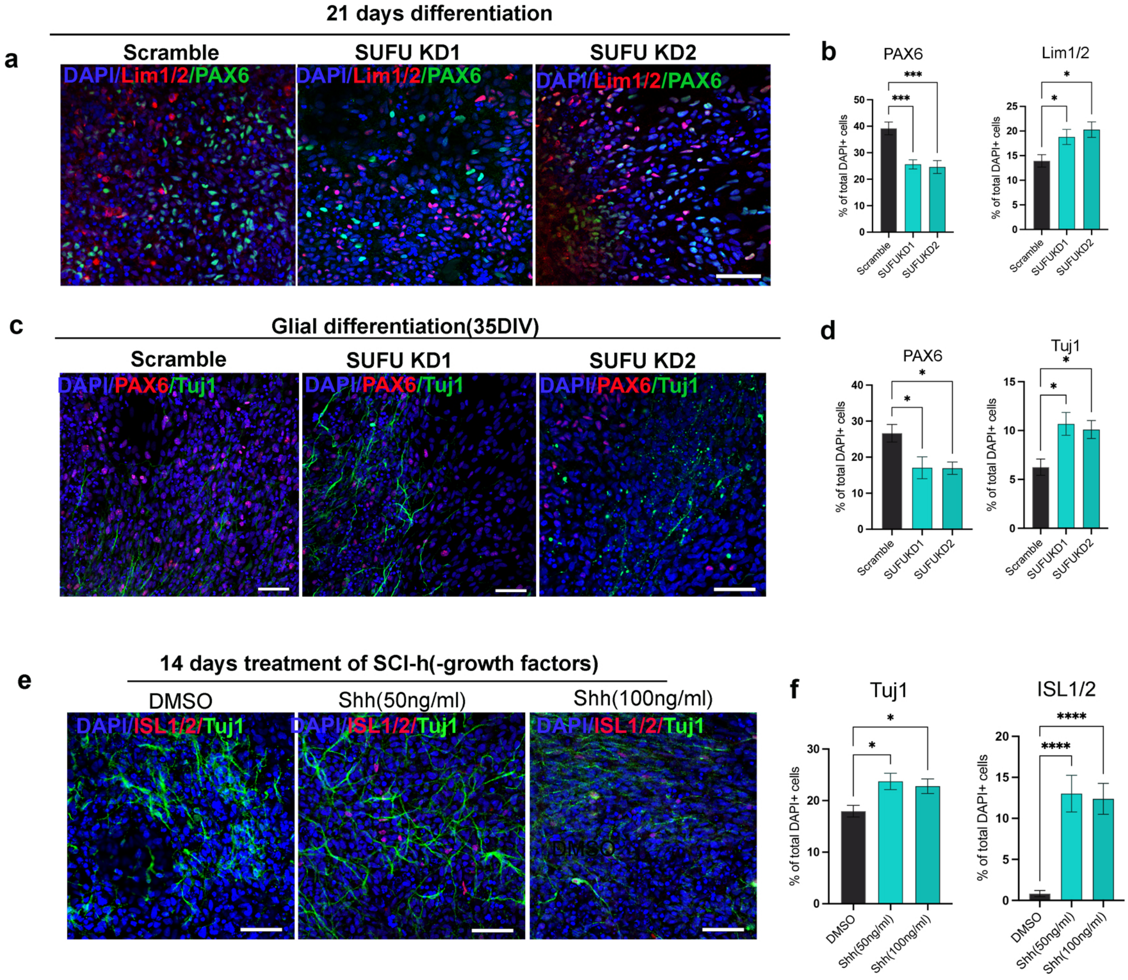

Supplementary Figure 2. Increased SHH activity leads to altered differentiation status.(a) Representative immunofluorescence images showing PAX6 and Lim1/2 expression in Scramble and *SUFUKD* hNPCs after 21 days of differentiation. The white box indicates the magnified view with the respective markers (Scale bar = 50 μm). Nuclei were stained with DAPI. **(b)** Quantification of PAX6 and Lim1/2 expression from (a). **(c)** Representative immunofluorescence images showing PAX6 and Tuj1 expression in Scramble and SUFU KD hNPCs after 3 weeks of differentiation in glial differentiating medium (Scale bar = 50 μm). **(d)** Quantification of PAX6 and Tuj1 expression from (c). **(e)** Representative immunofluorescence images showing ISL1/2, Tuj1, and nuclei marker DAPI expression in hNPCs treated with Shh recombinant protein (50 ng/ml and 100 μg/ml and homogenate (100 μg/ml) from the injured spinal cord (SCI-h) after 14 days of treatment (Scale bar = 50 μm). **(f)** Quantification of ISL1/2 and Tuj1 expression from (e). Student t-test. All data are expressed as mean ± SEM. *p<0.01, ***p<0.01, ****p<0.001 versus scramble. Three independent experiments.

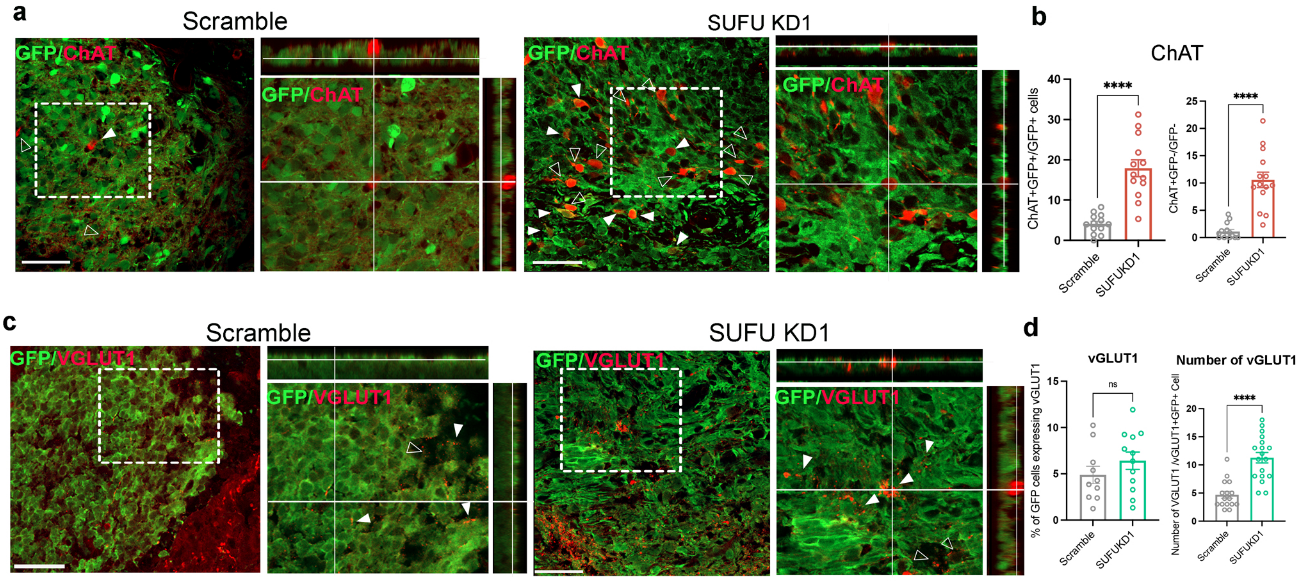

Supplementary Figure 3. Neuronal subtypes generated from Scramble and SUFUKD grafts. (a) Representative immunofluorescence images for GFP, ChAT(red) in sagittal sections of injured spinal cord with Scramble and *SUFUKD1* grafts at 2 month(2M post-graft. The empty arrow shows the indicated markers expression in GFP-positive cells The white arrow shows the indicated markers in GFP-negative cells. White box shows a zoomed-in view of the co-localization of indicated markers (Scale bar = 50 µm). **(b)**Quantification of the percentage of ChAT in grafts or non-grafts cells from(a). **(c)**Representative immunofluorescence images for GFP and vGLUT1(red) in sagittal sections with Scramble and *SUFUKD1* grafts at 2 month(2M) post-graft. The empty arrow shows the indicated markers expression in GFP-positive cells. The white arrow shows the indicated markers in GFP-negative cells. The white box shows a zoomed-in view of the co-localization of indicated markers. Scale bar = 50 µm. **(d)** Quantification of the percentage of vGLUT1 and number of vGLUT1/GFP cell in grafts from(c). Student t-test. All data are expressed as mean ± SEM. ****p<0.001 versus scramble. n = 5-6 rats per group, 4-5 sections/rats.

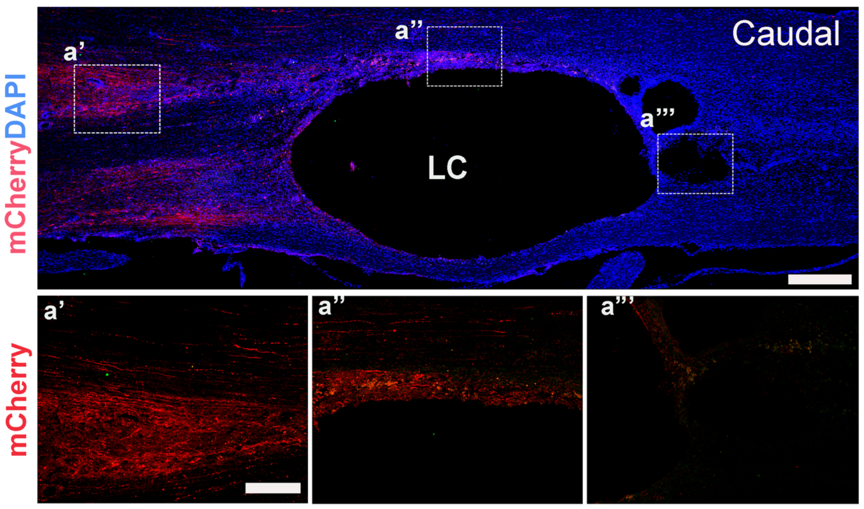

Supplementary Figure 4. AAV virus antegrade labelling of host connectivity in SCI lesion control. Sagittal section of lesion control showing antegrade, trans-synaptically traced host mCherry-expressing cells in the injured spinal cord without grafting. Scale bar, 500 µm. Inset, image showing injection sites in the brain region. Scale bar=100 µm. Inset (a-a''' and b-b'''), high-magnification view of the boxed area.

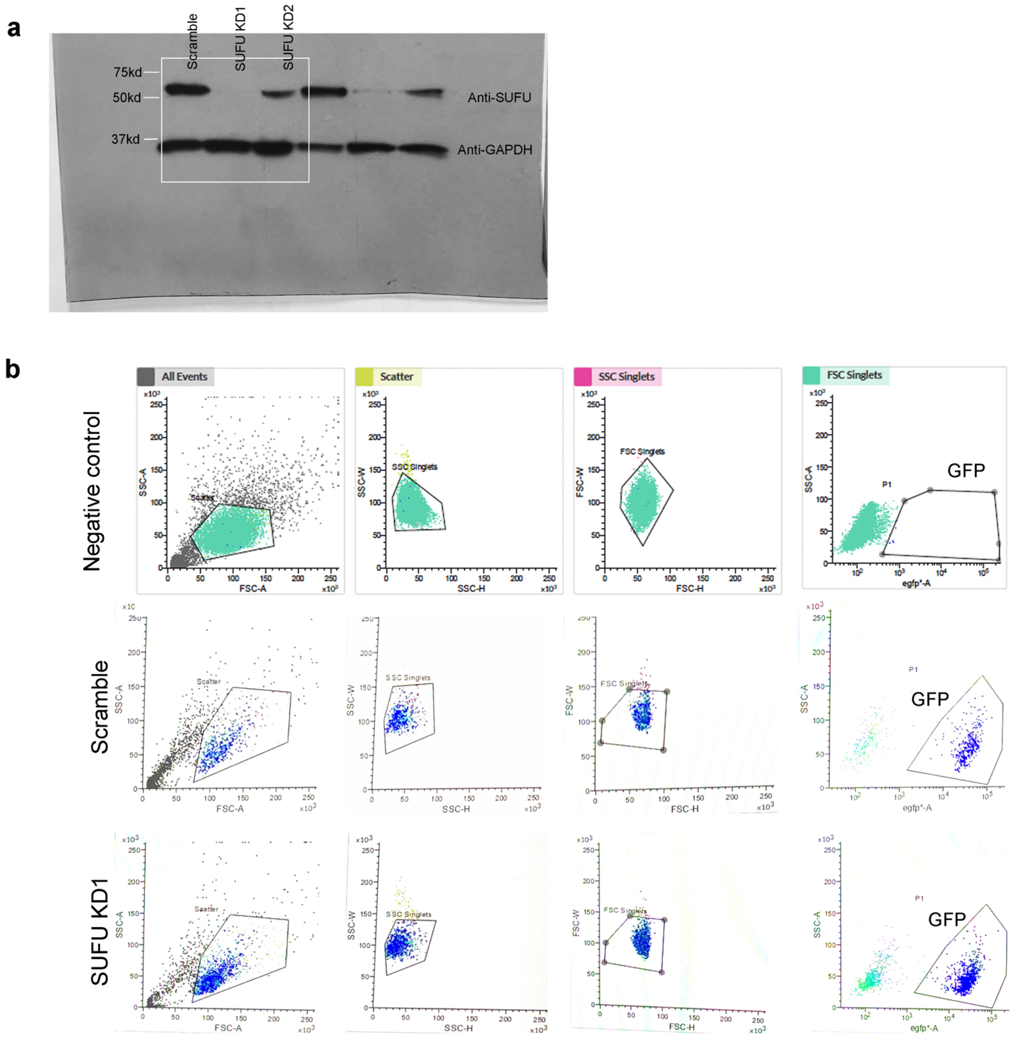

Supplementary Figure 5. **(a)**Raw data of uncropped western blots in Figure 1a. **(b)**FACS gating and sorting strategies for GFP cells of different treatments.

Supplementary Table 1: list of primary antibodies:

Antibody	Manufacturer	Catalog # / clone	Species
Sox10	R & D systems	AF2864	Goat
Sox2	R & D systems	MAB2018	Mouse
Sox2	Abcam	ab97959	Rabbit
Islet1/2	Developmental Studies Hybridoma Bank	39.4D5	mouse
Nkx6.1	Developmental Studies Hybridoma Bank	F55A12-c	mouse
HB9	Developmental Studies Hybridoma Bank	81.5C10	mouse
Olig2	Millipore	Ab9610	Rabbit
HuC/D	Invitrogen Antibodies	A-21271	Mouse
Pax6	Developmental Studies Hybridoma Bank	aa 1-223	mouse
Pax6	Life Technologies	1557865A	Rabbit
SUFU	Cell signaling	#2522	Rabbit
SHH	Developmental Studies Hybridoma Bank	5E1	Mouse
Caspase-3	Abcam	Ab2302	Rabbit
Tuj1	R & D systems	MAB1195	Mouse
CSPG	Sigma	CS-56	Mouse
MAP2	Abcam	ab32454	Rabbit
MAP2	Abcam	ab11267	Mouse
5-HT	ImmunoStar	20079	Goat
CaMKII	Abcam	ab52476	Rabbit
ChAT	Millipore	AB144P	Goat
GABA	Sigma	A2052	Rabbit
NF70 (Human)	Millipore	MAB5294	Mouse
NG2	Millipore	MAB2029	Mouse
GFAP	Dako	Z0334	Rabbit
GFAP	Abcam	Ab53554	Goat
Synaptophysin(human)	Novus Biologicals	NBP1-19222	Mouse
Synaptophysin	Developmental Studies Hybridoma	SV2A	Mouse

	Bank		
GFP	GeneTex	GTX13970	Chicken
MBP	Millipore	AB980	Rabbit

Supplementary Table 2: list of qPCR primers.

Gene symbol	**Forward**	**Reverse**
SUFU	TCGGCCTGAGTGATCTCTAT	CATTAACTCTGCGGGCCAT
SHH	CCGAGCGATTTAAGGAACTCACC	AGCGTTCAACTTGTCCTTACACC
NKX2.2	GAGTCACCGGACAATGACAA	GTCTGCGCCTTGGAGAAA
NKX6.1	GAAGAGGACGACGACTACAATAAG	CTGCTGGACTTGTGCTTCT
Olig2	CAGTGGCTTCAAGTCATCCT	GCTCCGGCTCTGTCATTT
PAX3	AAGAGGAAACAGCGCAGAAG	GGCCAGTTCCTCCCTAGTATAA
PTCH1	ACCGACACACACGACAATAC	ACAGTGGACTGCATGGTAATC
GLI1	GCAGTAAAGCCTTCAGCAATG	GCCAGGGAGCTTACATACATAC
GLI2	TGGCCGCTTCAGATGACAGATGTTG	CGTTAGCCGAATGTCAGCCGTGAAG
HIP1	GCACCACCTGTAGCATAGAAA	CCTGGGAGAGGAAGGAATTTG
ISLET1	TATCAGGTTGTACGGGATCAAA	CTACACAGCGGAAACACTCG
HB9	GCACCAGTTCAAGCTCAACA	CTTTTTGCTGCGTTTCCATT
Tuj1	TCAGCGTCTACTACAACGAGGC	GCCTGAAGAGATGTCCAAAGGC
MAP2	AAACTGCTCTTCCGCTCAGACACC	GTTCACTTGGGCAGGTCTCCACAA
SOX2	GCTGCAAAAGAGAACACCAATCCC	AAACTTCCTGCAAAGCTCCTACCG
SOX1	AGAACCGAATTCAGCCTGCATTCG	TTATCCCGGACTAAGTCGTAGTGG
GFAP	GAGAACCGGATCACCATTCC	CCCAGTCTGGAGCAACCTAC
NEUROD1	GGTGGTGCCTTGCTATTCTAA	AAAGCGTCTGAACGAAGGAG
PAX6	GCAACCTACGCAAGATGGCT	CTTTGCAGCTTCCGCTTCAGC
DCX	CCATTGATGGATCCAGGAAGAT	TGACAGACCAGTTGGGATTG
36B4	GTGATGTGCAGCTGATCAAGACT	GAAGACCAGCCCAAAGGAGA
