# Supplementary material for: Intrinsic and extrinsic actions of human neural progenitors with SUFU inhibition promote tissue repair and functional recovery from severe spinal cord injury
Source: NPJ Regen Med. 2024 Mar 22;9:13. doi: 10.1038/s41536-024-00352-4 (PMC10959923; doi:10.1038/s41536-024-00352-4)
Supplement: Supplementary file 2 — Reporting summary [file 41536_2024_352_MOESM2_ESM.pdf]

nature portfolio

Corresponding author(s): Jessica Liu

Last updated by author(s): 3rd Feb 2024

# Reporting Summary

Nature Portfolio wishes to improve the reproducibility of the work that we publish. This form provides structure for consistency and transparency in reporting. For further information on Nature Portfolio policies, see our Editorial Policies and the Editorial Policy Checklist.

## Statistics

For all statistical analyses, confirm that the following items are present in the figure legend, table legend, main text, or Methods section.

| n/a | Confirmed | |
|---|---|---|
| ☐ | ☒ | The exact sample size (*n*) for each experimental group/condition, given as a discrete number and unit of measurement |
| ☐ | ☒ | A statement on whether measurements were taken from distinct samples or whether the same sample was measured repeatedly |
| ☐ | ☒ | The statistical test(s) used AND whether they are one- or two-sided<br>*Only common tests should be described solely by name; describe more complex techniques in the Methods section.* |
| ☒ | ☐ | A description of all covariates tested |
| ☐ | ☒ | A description of any assumptions or corrections, such as tests of normality and adjustment for multiple comparisons |
| ☐ | ☒ | A full description of the statistical parameters including central tendency (e.g. means) or other basic estimates (e.g. regression coefficient) AND variation (e.g. standard deviation) or associated estimates of uncertainty (e.g. confidence intervals) |
| ☐ | ☒ | For null hypothesis testing, the test statistic (e.g. $F$, $t$, $r$) with confidence intervals, effect sizes, degrees of freedom and $P$ value noted<br>*Give P values as exact values whenever suitable.* |
| ☒ | ☐ | For Bayesian analysis, information on the choice of priors and Markov chain Monte Carlo settings |
| ☒ | ☐ | For hierarchical and complex designs, identification of the appropriate level for tests and full reporting of outcomes |
| ☒ | ☐ | Estimates of effect sizes (e.g. Cohen's $d$, Pearson's $r$), indicating how they were calculated |

*Our web collection on statistics for biologists contains articles on many of the points above.*

## Software and code

Policy information about availability of computer code

| | |
|---|---|
| Data collection | No software was used. |
| Data analysis | ImageJ and Zen offline were used for image analysis. GraphPad Prism 7 software were used for statistical analyses. |

For manuscripts utilizing custom algorithms or software that are central to the research but not yet described in published literature, software must be made available to editors and reviewers. We strongly encourage code deposition in a community repository (e.g. GitHub). See the Nature Portfolio guidelines for submitting code & software for further information.

## Data

Policy information about availability of data

All manuscripts must include a data availability statement. This statement should provide the following information, where applicable:

- Accession codes, unique identifiers, or web links for publicly available datasets
- A description of any restrictions on data availability
- For clinical datasets or third party data, please ensure that the statement adheres to our policy

The data that support the findings of this study are available from the corresponding author upon request.

## Research involving human participants, their data, or biological material

Policy information about studies with human participants or human data. See also policy information about sex, gender (identity/presentation), and sexual orientation and race, ethnicity and racism.

| | |
|---|---|
| Reporting on sex and gender | N/A |
| Reporting on race, ethnicity, or other socially relevant groupings | N/A |
| Population characteristics | N/A |
| Recruitment | N/A |
| Ethics oversight | N/A |

Note that full information on the approval of the study protocol must also be provided in the manuscript.

# Field-specific reporting

Please select the one below that is the best fit for your research. If you are not sure, read the appropriate sections before making your selection.

☒ Life sciences ☐ Behavioural & social sciences ☐ Ecological, evolutionary & environmental sciences

For a reference copy of the document with all sections, see nature.com/documents/nr-reporting-summary-flat.pdf

# Life sciences study design

All studies must disclose on these points even when the disclosure is negative.

| | |
|---|---|
| Sample size | P < 0.05 was determined to be significant. For any quantification in vivo, we used a sample N sufficient to yield data regarding the experimental question, never less than n=5 per group. For any quantification in vitro, we used at least three independent experiments, n>3/group. |
| Data exclusions | We excluded 6 rats in from behavioral tests, not developing phenotypes after spinal cord injury or did not wake up from anesthesia after surgery and died after the third surgery. |
| Replication | At least three replications |
| Randomization | Subjects were randomly assigned to each the experimental or control groups |
| Blinding | The experimenter was not blind to the identity of the animals during virus injections. Two investigators blinded to group for behavioral tests. |

# Reporting for specific materials, systems and methods

We require information from authors about some types of materials, experimental systems and methods used in many studies. Here, indicate whether each material, system or method listed is relevant to your study. If you are not sure if a list item applies to your research, read the appropriate section before selecting a response.

## Materials & experimental systems

| n/a | Involved in the study | |
|---|---|---|
| ☐ | ☒ | Antibodies |
| ☐ | ☒ | Eukaryotic cell lines |
| ☒ | ☐ | Palaeontology and archaeology |
| ☐ | ☒ | Animals and other organisms |
| ☒ | ☐ | Clinical data |
| ☒ | ☐ | Dual use research of concern |
| ☒ | ☐ | Plants |

## Methods

| n/a | Involved in the study | |
|---|---|---|
| ☒ | ☐ | ChIP-seq |
| ☐ | ☒ | Flow cytometry |
| ☒ | ☐ | MRI-based neuroimaging |

## Antibodies

| | |
|---|---|
| Antibodies used | listed all in supplementary table S1 |

| | |
|---|---|
| Validation | Sox10 R & D systems AF2864 Goat<br>Sox2 R & D systems MAB2018 Mouse<br>Sox2 Abcam ab97959 Rabbit<br>Islet1/2 Developmental Studies Hybridoma Bank 39.4D5 mouse<br>Nkx6.1 Developmental Studies Hybridoma Bank F55A12-c mouse<br>HB9 Developmental Studies Hybridoma Bank 81.5C10 mouse<br>Olig2 Millipore Ab9610 Rabbit<br>HuC/D Invitrogen Antibodies A-21271 Mouse<br>Pax6 Developmental Studies Hybridoma Bank aa 1-223 mouse<br>Pax6 Life Technologies 1557865A Rabbit<br>SUFU Cell signaling #2522<br>Rabbit<br>SHH Developmental Studies Hybridoma Bank 5E1 Mouse<br>Caspase-3<br>Abcam Ab2302 Rabbit<br>Tuj1 R & D systems MAB1195 Mouse<br>CSPG Sigma CS-56<br>Mouse<br>MAP2 Abcam ab32454 Rabbit<br>MAP2 Abcam ab11267 Mouse<br>5-HT ImmunoStar 20079 Goat<br>CaMKII Abcam ab52476 Rabbit<br>ChAT Millipore AB144P Goat<br>GABA Sigma A2052 Rabbit<br>NF70 (Human) Millipore MAB5294 Mouse<br>NG2 (Human) Millipore MAB2029 Mouse<br>GFAP Dako Z0334 Rabbit<br>GFAP Abcam Ab53554 Goat<br>Synaptophysin(human) Novus Biologicals NBP1-19222 Mouse<br>Synaptophysin Developmental Studies Hybridoma Bank SV2A Mouse<br>GFP GeneTex GTX13970 Chicken<br>MBP Millipore AB980 Rabbit |

## Eukaryotic cell lines

Policy information about cell lines and Sex and Gender in Research

| | |
|---|---|
| Cell line source(s) | H9 ESCs (WA-09 (H9)and HES2 were provided from WiCell Research Institute . IMR90 were provided from Dr. KO LAI(CityU), who purchased from WiCell. |
| Authentication | All pluripotent cell lines were authenticated by their providers and also validated by KLF4, OCT4 and SOX2 expressions (Immunostaining and qPCR) before neural induction. |
| Mycoplasma contamination | All pluripotent cell lines were confirmed negative for mycoplasma contamination by routine tests. |
| Commonly misidentified lines (See ICLAC register) | These cell lines are not in the database, as of version 12. |

## Animals and other research organisms

Policy information about studies involving animals; ARRIVE guidelines recommended for reporting animal research, and Sex and Gender in Research

| | |
|---|---|
| Laboratory animals | Sprague-Dawley male rats were provided by the Centre for Comparative Medicine Research, Li Ka Shing Faculty of Medicine, The University of Hong Kong. |
| Wild animals | N/A |
| Reporting on sex | Male |
| Field-collected samples | N/A |
| Ethics oversight | All animal protocols were approved by the Committee on the use of live animals in Teaching & Research of The University of Hong Kong. |

Note that full information on the approval of the study protocol must also be provided in the manuscript.

## Plants

| | |
|---|---|
| Seed stocks | N/A |
| Novel plant genotypes | N/S |
| Authentication | N/A |

## Flow Cytometry

### Plots

Confirm that:

☒ The axis labels state the marker and fluorochrome used (e.g. CD4-FITC).

☒ The axis scales are clearly visible. Include numbers along axes only for bottom left plot of group (a 'group' is an analysis of identical markers).

☒ All plots are contour plots with outliers or pseudocolor plots.

☒ A numerical value for number of cells or percentage (with statistics) is provided.

### Methodology

| | |
|---|---|
| Sample preparation | Cells were harvested and dissociated with Accutase (Gibco, #2310195) at 37°C for 5 mins to yield a single cell suspension, and finally resuspended in FACS buffer (1X PBS (Gibco, 70013032) + 0.4% BSA (Sigma, A7638)) after twice of wash steps. |
| Instrument | BD FACSMelody |
| Software | BD FACSChorusTM<br>https://cpos.hku.hk/content/uploads/2021/02/Melody-Standard-Operation-Protocol_-Basic-Operation.pdf |
| Cell population abundance | Negative control: 0.04%<br>Plenti-gfp: 1.74%<br>Scr-GFP: ~15%<br>SUFUKD1-GFP 30.85% |
| Gating strategy | The single cells were gated by the steps of FSC-A/SSC-A, SSC-H/SSC-W, FSC-H/FSC-W. Then, single cells were visualized by the EGFP-A/SSC-A. The EGFP positive and negatives were distinguished by fluorescence intensity and showed as two different cell group. The cells with high fluorescence intensity (>104) were recognized as EGFP positive cells and collected. |

☒ Tick this box to confirm that a figure exemplifying the gating strategy is provided in the Supplementary Information.
